# In silico insights into the design of novel NR2B-selective NMDA receptor antagonists: QSAR modeling, ADME-toxicity predictions, molecular docking, and molecular dynamics investigations

**DOI:** 10.1186/s13065-024-01248-6

**Published:** 2024-07-31

**Authors:** Mohamed El fadili, Mohammed Er-rajy, Somdutt Mujwar, Abduljelil Ajala, Rachid Bouzammit, Mohammed Kara, Hatem A. Abuelizz, Sara Er-rahmani, Menana Elhallaoui

**Affiliations:** 1 LIMAS Laboratory, Faculty of Sciences Dhar El Mahraz, Sidi Mohamed Ben Abdellah University, Fez, 30000 Morocco; 2 Chitkara College of Pharmacy, Chitkara University, Rajpura, Punjab 140401 India; 3 Department of chemistry, Faculty of physical sciences, Ahmadu Bello University, Zaria, Nigeria; 4 Engineering Laboratory of Organometallic, Molecular Materials and Environment (LIMOME), Faculty of Sciences Dhar El Mahraz, Sidi Mohamed Ben Abdellah University, Fez, 30000 Morocco; 5 Laboratory of Biotechnology, Conservation and Valorization of Naturals Resources, Faculty of Sciences Dhar El Mahraz, Sidi Mohamed Ben Abdellah University, Fez, 30000 Morocco; 6 Department of Pharmaceutical Chemistry, College of Pharmacy, King Saud University, Riyadh, Saudi Arabia; 7 Dipartimento di Chimica, Università di Torino, Torino, 10125 Italy

**Keywords:** NMDA receptor, Analgesic activity, Molecular modeling, Neuropathic pain, MD

## Abstract

Based on a structural family of thirty-two NR2B-selective N-Methyl-D-Aspartate receptor (NMDAR) antagonists, two phenylpiperazine derivatives labeled C37 and C39 were conceived thanks to molecular modeling techniques, as novel NMDAR inhibitors exhibiting the highest analgesic activities (of pIC_50_ order) against neuropathic pain, with excellent ADME-toxicity profiles, and good levels of molecular stability towards the targeted protein of NMDA receptor. Initially, the quantitative structure-activity relationships (QSARs) models were developed using multiple linear regression (MLR), partial least square regression (PLSR), multiple non-linear regression (MNLR), and artificial neural network (ANN) techniques, revealing that analgesic activity was strongly correlated with dipole moment, octanol/water partition coefficient, Oxygen mass percentage, electronegativity, and energy of the lowest unoccupied molecular orbital, whose the correlation coefficients of generated models were: **0.860**, **0.758**, **0.885** and **0.977**, respectively. The predictive capacity of each model was evaluated by an external validation with correlation coefficients of **0.703**, **0.851**, **0.778**, and **0.981** respectively, followed by a cross-validation technique with the leave-one-out procedure (CVLOO) with Q^2^_cv_ of **0.785**, more than Y-randomization test, and applicability domain (AD), in addition to Fisher’s and Student’s statistical tests. Thereafter, ten novel molecules were designed based on MLR QSAR model, then predicted with their ADME-Toxicity profiles and subsequently examined for their similarity to the drug candidates. Finally, two of the most active compounds (C37 and C39) were chosen for molecular docking and molecular dynamics (MD) investigations during 100 ns of MD simulation time in complex with the targeted protein of NMDA receptor (5EWJ.pdb).

**Supplementary Information:**

The online version contains supplementary material available at 10.1186/s13065-024-01248-6.

## Introduction

N-methyl-D-aspartate (NMDA) receptors are part of ionotropic receptors physiologically activated by glutamate and glycine, which play an essential role in learning, memory, and synaptic plasticity in the central nervous system (CNS). They are mainly known for their permeability to divalent cations of calcium (Ca^2+^) and monovalent cations of potassium (K^+^) and sodium (Na^+^) in membrane cells, due to their structural nature as a tetrameric combination of two GluN1subunits linked to glycine and two GluN2 subunits fixed to glutamate sites, in which the distribution of each NMDA subunit is closely associated with neuronal plasticity and synaptic deficiencies [1]. Recently, it was discovered as a potential therapeutic target for neurological and mental diseases, including memory impairments, chronic pain, schizophrenia, Parkinson’s, and Alzheimer’s disorders [2–5]. In this respect, the discovery of powerful NMDA receptor antagonists is an absolute necessity for treating this class of neurodegenerative disorders, which requires a selective activation of each blocked receptor which occurs more frequently for older cells than younger ones.

Nowadays, the computer-assisted drug design (CADD) approach based on in silico techniques has taken on enormous importance in the pharmaceutical sector, aimed at discovering new drug candidates [6–8]. It has been widely reported in the literature to save the time and costs of experimentation before any pre-clinical and clinical tests. However, in vivo and in vitro tests remain strongly recommended for each new compound designed based on in silico studies [9–12]. Many scientific articles were focused on NMDA receptors as a major neurotransmitter in the human brain, closely involved in the excitotoxicity process responsible for the pathophysiology of various diseases, to concept the new competitive and non-competitive NMDA receptor antagonists, which could be recommended as an effective treatment for several neurological impairments such as Alzheimer’s, cerebral vascular accidents, and chronic pain [13–16].

The present work aims to concept novel NR2B-selective NMDAR antagonists using in-silico techniques based on computer molecular modeling, including QSAR modeling, ADME-Tox predictions, molecular docking, and molecular dynamics investigations.

In the first stage, QSAR technique was carried out to a structural class of thirty-two selective inhibitors of NMDA receptor, acting as analgesic agents against neuropathic pain [5, 17], in which four QSAR models were generated using MLR, PLSR, MNLR, and ANN techniques to predict the linear and nonlinear relationship between various molecular descriptors of NR2B-selective NMDAR antagonists and their analgesic activities of pIC_50_ order [18, 19]. The developed models were applied on a training set of twenty-six molecules and then validated on a test set of six molecules [20]. Thereafter, ten novel molecules (C33 to C42) were designed based on the most active compound (C22) and predicted in humans by their absorption, distribution, metabolism, excretion, and toxicity (ADMET), then examined for their similarity to the drug candidates [21]. In the second stage, two novel-designed molecules labeled C37 and C39, were chosen for molecular docking to study their inhibition mechanisms towards NMDA receptor, encoded in protein data bank by 5EWJ.pdb [22]. In the final stage, C37 and C39 molecules, which were predicted to have greater analgesic activity than the most active compound C22, were chosen for the molecular dynamics (MD) technique to test the thermodynamic stability of each (ligand-protein) complex [23, 24], which was equally compared to the original compound (C22) over 100 nanoseconds of MD simulation time after being complexed to the same protein target.

## Materials and methods

### Experimental database

A structural family of 32 NMDA receptor antagonists, which were successfully examined using experimental in vivo and in vitro assays to block the binding of [3 H]-ifenprodil to rat brain membranes as authored by Kosuke Anan, et al. [17], was chosen as an appropriate database to concept new active molecules with the highest inhibitory activities using molecular modeling techniques, where the analgesic activities against neuropathic pain of the original molecules (C1 to C32) were expressed on a decimal logarithm scale (pIC_50_ = - log_10_IC_50_), as presented in Table 1.

**Table 1:**
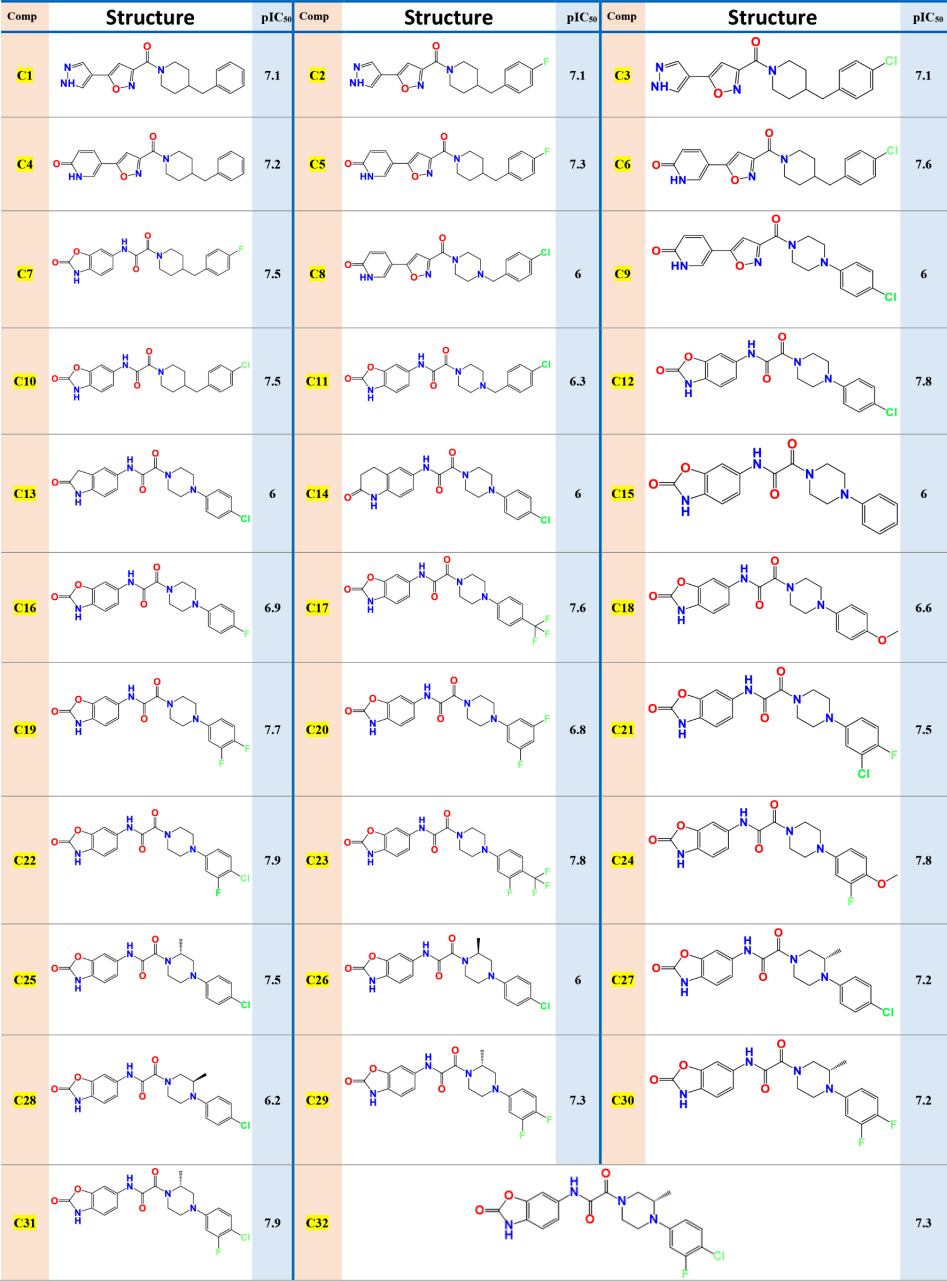
Studied molecules and their observed activities pIC_50_

### Molecular descriptors calculation

To establish a reliable QSAR model, we have calculated a variety of molecular descriptors as classified in Table 2, where the thermodynamic and physicochemical descriptors were calculated by MM2 method using ChemBio3D software [25], the constitutional descriptors were calculated by ACD/chemsketch software [26], at the same time the geometry of the studied compounds was optimized using the density functional theory (DFT) with the B3LYP functional [27], combined with the 6-31G + G(d, p) basis, thus the quantum descriptors were calculated with the help of Gaussian 09 package software [28].

**Table 2:** List of different calculated descriptors

Type of descriptors	Name of descriptors
Quantum descriptors	HOMO and LUMO Energies (E homo, E lumo) - Hardness (ƞ) Dipole moment (µ) - Electronegativity (χ) - Gap Energy (E gap)
Constitutional descriptors	% C - % H - % O
Physicochemical descriptors	Octanol-water partition coefficient (Log P) - Density (d)
Thermodynamic descriptors	Kinetic Energy (K) - Potential Energy (P) - Total Energy (T)

### Statistical methods

To generate the mathematical models that exhibit the quantitative relationships between the biological activity and the calculated molecular descriptors, different statistical methods were used such as Multiple Linear Regression (MLR), Partial Least Squares Regression (PLSR), Multiple Non-Linear Regression (MNLR) and Artificial Neural Network (ANN).

As a first step, the number of molecular descriptors was minimized using the Principal Component Analysis (PCA) by XLSTAT 2014 software, as a very important step to make the studied information less redundant, because it serves to reduce the size of the original variables that are correlated with each other into some synthetic variables that are independent from one another, called the principal components or factorial axes [29–31].

As a second step, the linear variation of these independent variables (uncorrelated descriptors) with the dependent variable (analgesic activity), was tested using MLR method with stepwise selection. In this case, where there are more predictors than observations and there is strong collinearity between these predictors, the PLS method is the best and it would be interesting to be able to study more precisely the quality of the model by minimizing the difference between the observed and calculated values [32]. Finally, the molecular descriptors used were chosen as input parameters in the multiple nonlinear regression (MNLR) and artificial neural network (ANN). All four models obtained were generated to predict the effects of these NMDA antagonists on the rat brain. Each tested model was treated according to the following statistical criteria: *R*, *R*^*2*^, and *R*^*2*^*adj* that must tend towards 1, which mean respectively: correlation coefficient, determination coefficient, and adjusted coefficient. Mean square error (*MSE*) should be minimal (tend towards 0), the Fisher value (*F*) must be inferior to the critical value of Fisher, and the probability value (*p-value*), it’s better to be under 5% for a 95% confidence level. To make these four QSAR models applicable, we have used the external validation technique on six new compounds constituting the test set. Also, we have performed the cross-validation method with the “leave-one-out” procedure, in order to examine the reliability of the developed models. So, the validity of the performance estimate will be obtained by performing this conventional procedure, which is realized by removing a single example from the training base, applied each time on the *N*-1 compounds [33]. In addition to cross-validation method, two other techniques are very important: the first one is “Y-randomization” technique; this sort of approaches is particularly appropriate when we are not sure of our model of the data generation process [34]. To do this, we were based on our full set of 32 compounds, after that we have searched randomly a new training test of 26 molecules to validate and ensure the security of the statistical tests [35].

### Drug likness and in-silico ADMET predictions

To examine the drug likness of novel chemical compound, the physicochemical feautures must satisfy at least two of Lipinski’s five rules, with an acceptable ADMET profile, and good oral biovailability by the human body, without any adverse toxic effects on the human body. For this purpose, SwissADMET, pKCSM, and CLC Drug Discovery servers were carefully used to test the physicochemical and pharmacokinetic profiles [36, 37] of nine chemical compounds designed based on QSAR results.

### Molecular docking modeling

Molecular docking is often used in computational chemistry to accelerate drug discovery at early stages [9]. For this project, this molecular modeling technique is based on the cell key phenomenon, where the best position of the ligand or drug candidate (the agent) is the key that can open the cell (or protein) to have finally a more stable complex by energetic order [38].

The crystal structure of amino-terminal domains of the NMDA receptor subunit GluN1 and GluN2B in complex with ifenprodil, coded in protein data bank by 5EWJ.pdb (https://www.rcsb.org/structure/5EWJ) was chosen as the targeted protein of NMDA receptor, in which can complex the inhibitors studied [39]. The responsible protein was discovered using X-ray diffraction method, in machine simulation with a resolution equal to 2.77 Å [40]. Then, it was prepared by removing all the water molecules bound, the sodium atom, and all the suspended ligands were removed, while adding the polar hydrogens using the Discovery Studio software, for the reason that the cavity method works best [41]. All this is to indicate the map coordinates of the co-crystallized ligand. After the preparation of the protein and quoting its active site, we have launched the docking calculation in AutoDock 4.2 program. In this way we have docked in the protein receptor three molecules already optimized by the DFT theory (output file): the first one is noted C22, having the greatest analgesic activity against neuropathic disease of pIC50 = 7.9, and the second ones are the new predicted molecules (C37 and C39) to compare their mechanisms of inhibition towards the most active compound. With the help of algorithm AUTOGRID We were able to centralize the grid boxes on (86.63 Å, -7.51 Å, -64.01 Å) by putting the sizes: 110, 110, and 110 in their three-dimensional structure, and running 10 genetic algorithms, with a total of two million five hundred thousand evals. At the end, we have got the strongest complex out of fifty conformations obtained [42], and we have visualized the 2D and 3D interactions of the protein-ligand by discovery studio 2020 [43].

### Molecular dynamics simulation

To validate the thermodynamic stability of ligands C37, C39, and standard drug C22 with the NMDA receptor for their analgesic effect were shortlisted for performing MD simulation based upon their docking score, physicochemical analysis and observed chemical interactions with the target receptors. All the above said MD simulations were executed for a time period of 100 ns by using Desmond module of Schrodinger’s Maestro software [44]. Addition of explicit solvent molecules followed by their neutralization by adding the respective ions. The steepest-descent algorithm was used to relax the system and eliminate any steric clashes or poor contacts within atoms in order to minimize the system’s energy. Using a short series having low temperature with constant pressure (NPT) simulations, the system was brought to equilibrium. Positional constraints are applied to the system in addition to a progressive increase in temperature [45–47]. This makes it more likely that the system will be in a stable, balanced state prior to the simulation, in order to get the appropriate outcomes, the simulation is performed for 100 ns while taking into account the system’s energies, atom positions, and RMSD values. This aids in comprehending the system’s dynamic behavior and provides long-term intuitions on the complex’s structure and functional stability [24, 48–50].

## Results and discussions

### Statistical database

To establish the QSAR mathematical models, we have selected a set of 32 compounds from recently published work of K.Anan, et al., including NR2B-selective antagonists that Inhibit binding of [3 H]-ifenprodil to rat brain membranes [17]. Therefore, the database will be represented as a matrix of 32 rows (active molecules) and 14 columns (molecular descriptors) as illustrated in Table S1. Then, we worked randomly to divide this complete set into two subsets: the first one (training set) includes 26 compounds that are used to build the model and the second one (test set) includes 6 compounds to validate this established model.

### Principal component analysis

To reduce the size of the basic data, we have applied the principal component analysis method, calculating a number of linear combinations of the original variables such a way to summarize the data with minimal loss of information, focusing on the correlation matrix given in Table S2 [51].

**Fig. 1:**
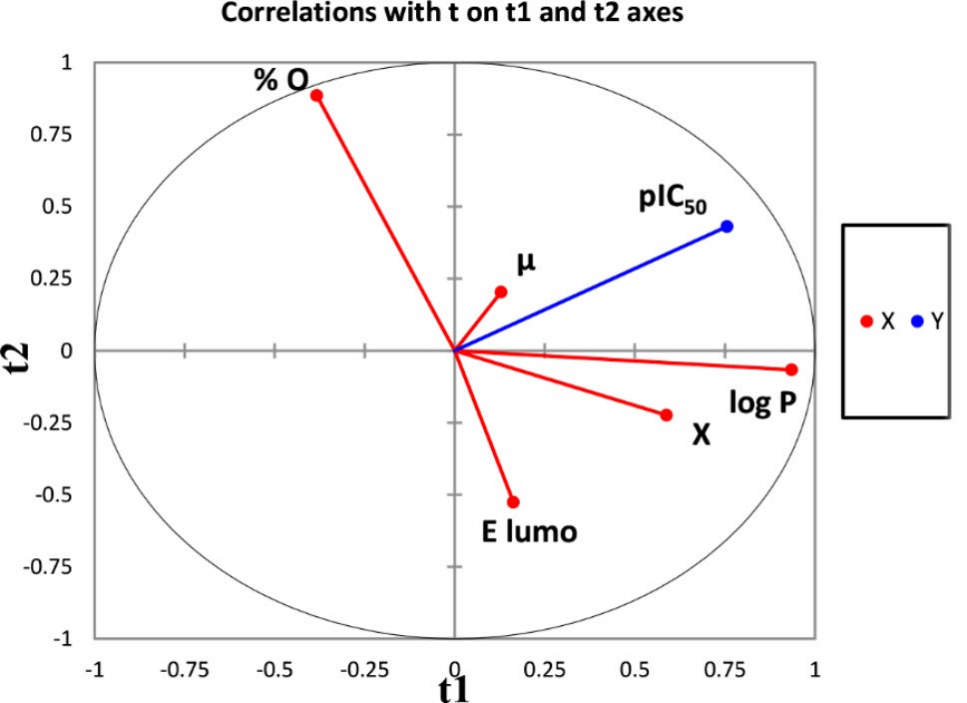
The projection of the uncorrelated variables on the plane of the first two principal components t1 and t2

According to the correlation matrix, the variables that have a correlation coefficient absolutely higher than **90%** are strongly correlated. So, we have found 9 following correlations:

First, the density is strongly and negatively correlated with % C and % H of -94.5% and − 96.1% respectively, also a strong positive correlation of 94.7% between % C and % H, this means that the three are correlated with each other several times. Second, it was observed that: the potential energy (P), kinetic energy (K) and the total energy (T), are totally correlated because the correlation coefficient is equal to 1 in absolute value. Third, E Homo, E gap and hardness are strongly correlated with each other, such as: *r*(E homo, E gap)= -90.2%, *r*(E homo, ƞ)= -90.4% and *r*(ƞ, E gap) = 99.8%. Finally, we have dropped these nine descriptors because every three variables share the same information, as they are largely correlated with each other. Thus, we have limited our study to five major uncorrelated descriptors pictured in Fig. 1.

**Fig. 2:**
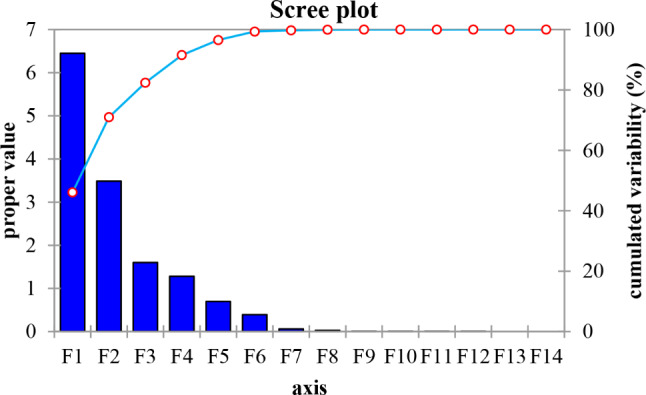
The explanatory power of the principal components

These descriptors are projected on the first two principal components t1 and t2. Since, 46.074% of the studied variables are explained by the first principal component, 24.901% of the variables contribute to the construction of the second and only 11.441% of the variables contribute to the construction of the third principal component. Therefore, we can propose that only the first two principal components are sufficient to obtain a good explanation of the data, because the variability explained by the first two principal components is: 70.97% of the total variability, as illustrated in Fig. 2.

### Multiple linear regression

The Multiple Linear Regression is a statistical technique for estimating the relationship between more than two variables which have cause-effect relations [52]. For that, we have applied this technique on the training test including twenty-six observations (*N* = 26) and we have found the best following QSAR model as illustrated in the following equation:

1 $$\begin{aligned}\text{pIC}_{50}&=-5.031+42.028\ast\text{E\;lumo}+65.706\ast{\chi}-0.228\ast{\mu}\\&\;+1.308\ast \text{Log\;P}+0.244\ast\%\text{O} \end{aligned}$$

This established model shows that biological activity, is a quantitative variable significantly influenced by the following five descriptors: E lumo, χ, µ, Log P and % O, because the probability corresponding to the slope of each variable is less than 5% as noted in Table 3.

**Table 3:** Significance test of the slopes

Source	Value	t	Pr > |t|	Lower Terminal (95%)	Higher Terminal (95%)
Constant	-5.031	-2.341	**0.030**	-9.514	-0.549
E lumo	42.028	2.174	**0.042**	1.706	82.349
X	65.706	3.567	**0.002**	27.277	104.134
µ	-0.228	-3.353	**0.003**	-0.371	-0.086
Log P	1.308	9.038	**< 0.0001**	1.006	1.610
% O	0.244	5.974	**< 0.0001**	0.159	0.329

**Fig. 3:**
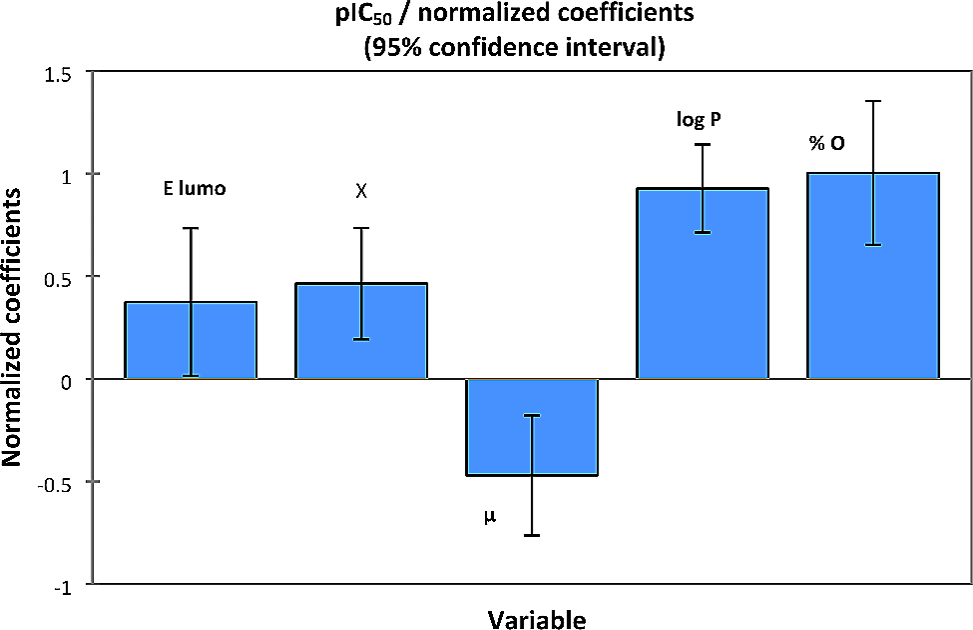
Degree of influence of the descriptors on the activity

To know, all the variables have a positive weight on the biological activity except the dipole moment, as shown in Fig. 3, because each increase in activity is accompanied by an increase of 42.028 kcal/mol in the energy of the lowest unoccupied molecular orbital, 65.706 in the electronegativity of the compound, 1.308 log P (Hight lipophilicity), and 0.244% of oxygen, but results in a decrease of 0.228 in the dipole moment. For a 95% confidence interval, the null hypothesis (*H0*) posed by the Fisher statistical test is rejected, because the calculated Fisher value (*F* = 24.664) is much higher than the critical value: [*F* (26, 5) = 2.59, *p* < 0.0001], as simplified by the one ANOVA test (Table 4). So, the variance is homogeneous between the dependent variable (pIC_50_) and the five explicative variables. In addition, the determination, correlation, and adjustment coefficients of *R* = 0.928, R²=0.860 and R²adjusted = 0.826, show a strong relationship between descriptors and answers. So, the first QSAR model obtained by MLR has good predictive competence, with a minimal standard error (*RMSE* = 0.272).

**Table 4:** Analysis of variance (ANOVA 1)

Source	DDL	Total square	Mean square	F	Pr > F
Model	5	9.103	1.821	**24.664**	**< 0.0001**
Error	20	1.476	0.074		
Adjusted total	25	10.579			

### Partial least squares regression (PLSR)

To model a linear relationship between a set of predictors and a response variable, so that the variance is minimal, we have applied the partial least squares regression technique, in a way that solves linear regression problems and to have finally a good quality of adjustment, and a good predictive power [53, 54]. The results of this regression technique yield the second QSAR model given in the following Eq. 2:

2 $$\begin{aligned}\text{pIC}_{50}&=-3.696-20.573\ast\text{E\;lumo}+37.531\ast\text{X}+2.353\text{E-02}\ast{\mu}\\&\;+1.229\ast \text{Log\;P}+0.121\ast\%\text{O}. \end{aligned}$$

This model generated by the PLSR technique, is also applied on the same training set of twenty-six observations (*N* = 26) and given by the following statistical criteria: the standard error (*RMSE* = 0.314) is minimal, the determination and correlation coefficients which are respectively: *R* = 0.870 and *R²*=0.758, show that there is a good quality of adjustment and a good predictive power of the model made by the regression on the partial least squares.

### Multiple non-linear regression (MNLR)

Non-linear regression analysis is a type of regression analysis in which the data are modeled by a non-linear combination of several independent variables [55]. To realize this technique, we have used preprogrammed function of type:

3 $$\:Y=a0+{\sum\:}_{i=1}^{n}\left(ai*Xi+bi*{Xi}^{2}\right)$$

As: Y: the predicted biological activity (pIC_50_), Xi: the explicative variable, a0: the constant of the QSAR model, a.i.: the slope of each descriptor to one degree and bi: the slope of each descriptor of two degrees. This function presents the non-linear combination of the biological activity (pIC_50_) as a function of the five independent variables found previously by the linear model QSAR, based on the same training set of twenty-six observations (*N* = 26). So, the final results produce the third QSAR model as indicated in the following Eq. 4:

4 $$\begin{aligned}\text{pIC}_{50}&=23.360+64.956\ast\text{E\;lumo}-354.817\ast\text{X}-0.360\ast{\mu}\\&+3.680\ast\log\;\text{P}-0.166\ast\%\;\text{O}+297.460\ast\;\text{E\;lumo}^{2}\\&+1599.438\ast\text{X}^{2}+2.258\text{E-2}\ast\mu^{2}\\&-0.550\ast\log\;\text{P}^{2}+1.461\text{E-2}\ast\%\text{O}^{2}\end{aligned}$$

This mathematical model is defined by a good coefficient of determination (*R* = 0.941) and a good correlation coefficient (*R*^*2*^ = 0.885), in addition the model Root Mean Square Error is minimal (*RMSE* = 0.285), this means that the last QSAR model generated by the MNLR technique has a good predictive competence and a strong non-linear relationship between descriptors and answer.

### Artificial neural network (ANN)

One of the most important steps in QSAR studies is to develop a non-linear relationship between activity and molecular descriptors, using the ANN method [56], which differs from the function of biological neurons in a number of parameters such as structures, layers, computational style, processing speed, connections, strength, storage and transmission of information [57]. In the present study, we have used three-layer neural networks, the input layer contains 5 neurons representing the selected descriptors, the hidden layer contains 3 neurons (H1, H2 and H3), and the output layer represents the observed activity values (pIC_50_). A parameter ρ serves a major role in the determination of the best ANN architecture, is used to identify the number of hidden neurons [58], which must be in the interval: 1 < *ρ* < 3 [59]. It’s given by the following Eq. 5:

5 $${\rho}=\frac{\:\text{T}\text{h}\text{e}\:\text{n}\text{u}\text{m}\text{b}\text{e}\text{r}\:\text{o}\text{f}\:\text{w}\text{e}\text{i}\text{g}\text{h}\text{t}}{\text{T}\text{h}\text{e}\:\text{n}\text{u}\text{m}\text{b}\text{e}\text{r}\:\text{o}\text{f}\:\text{c}\text{o}\text{n}\text{n}\text{e}\text{c}\text{t}\text{i}\text{o}\text{n}\text{s}\:\text{i}\text{n}\:\text{t}\text{h}\text{e}\:\text{N}\text{N}}$$

**Fig. 4:**
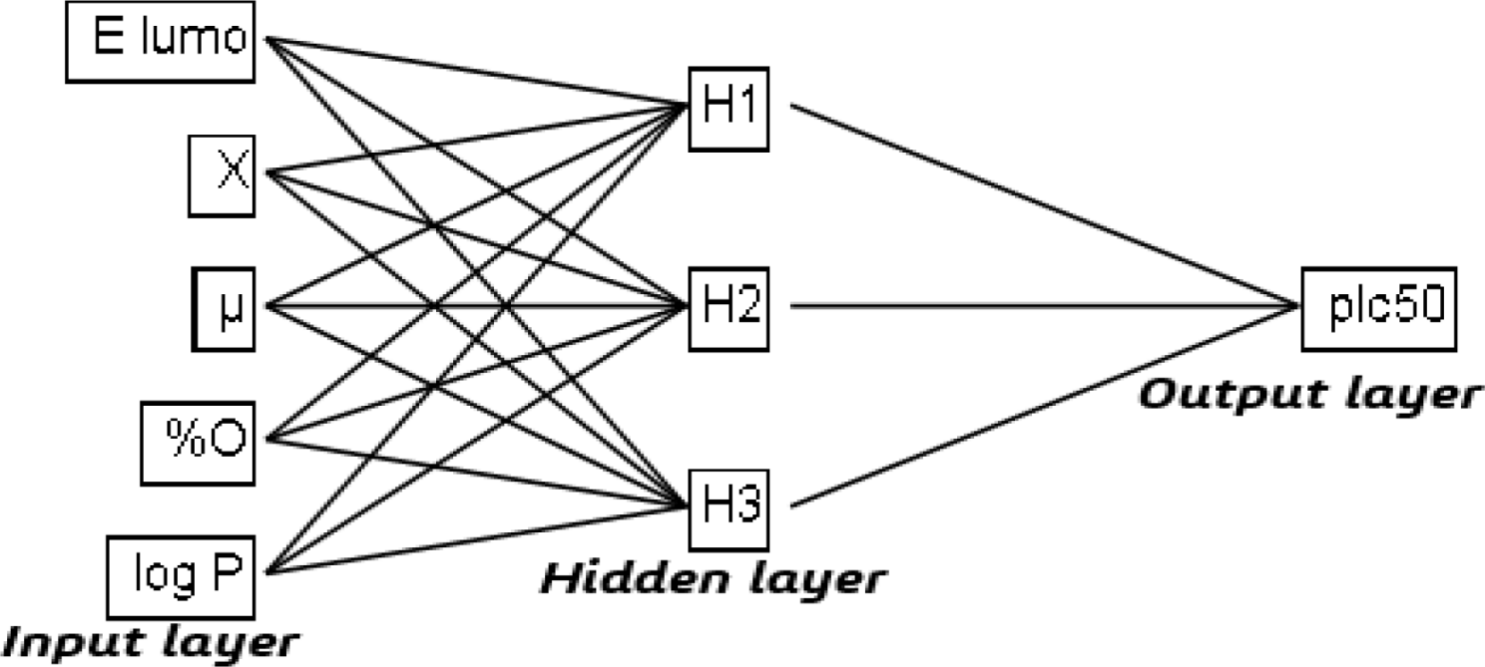
Schematic diagram of three-layer artificial neural network

So, the architecture of the Artificial Neural Networks used in this work is [5–3–1], as shown in Fig. 4.

The obtained results of the artificial neural network, using the JMP version 8.0 software, show a very good correlation of 97.7% between the observed and predicted values, with a minimal coded residual standard error equal to 0.189 and an external validation correlation coefficient of 98.1%, operating on 16 turns and 2.734 of over-adjustment penalty, with 75 maximum iterations, and 0.00001 of convergence criteria. This indicates that the descriptors selected by multiple linear regression, are relevant, and the model has a high statistical quality.

### Validation techniques

#### External validation

To evaluate the accuracy of four predictive models and ensure their generalizability, it is essential to validate them externally before they are applied in clinical practice [60]. For this purpose, we have tested the six new molecules constituting the test set on a training test basis and we have arrived at the results presented in Table 5.

**Table 5:** The results of external validation by the MLR, PLSR and MNLR methods

*N*°	Observed pIC_50_	pIC_50_ MLR	pIC_50_ PLSR	pIC_50_ MNLR
C4*	7.200	6.644	7.021	6.749
C19*	7.700	6.975	6.864	6.914
C24*	7.800	6.526	6.565	6.721
C26*	6.000	7.375	7.481	7.334
C28*	6.200	7.470	7.371	7.396
C30*	7.200	7.094	7.211	7.113

*Indicates the test set molecules

**Fig. 5:**
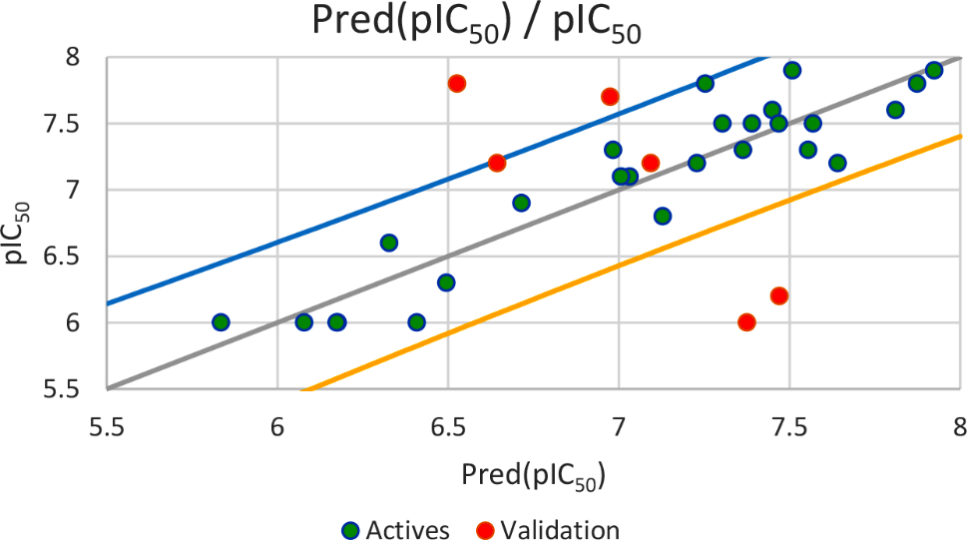
Correlation between the observed and predicted activities using MLR technique

**Fig. 6:**
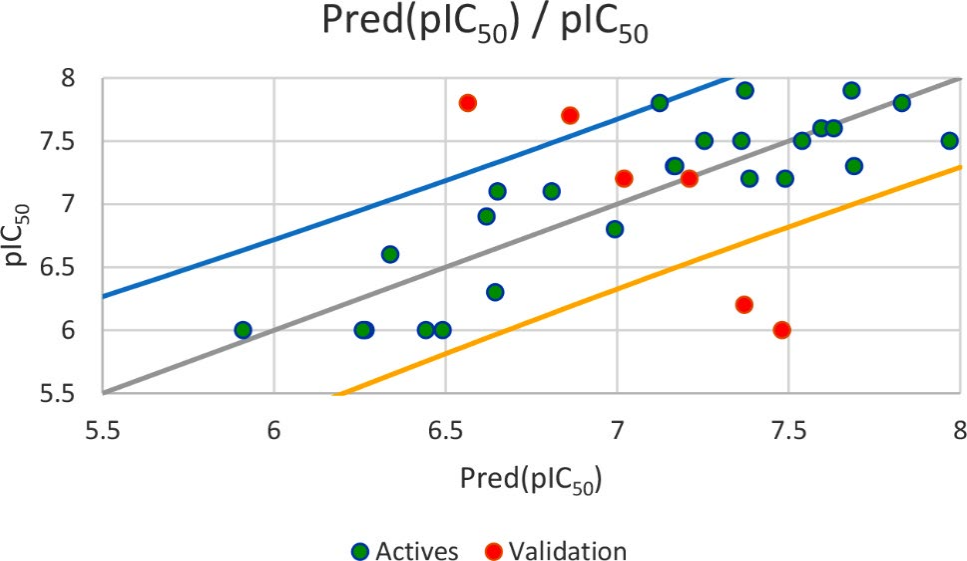
Correlation between the observed and predicted activities using PLSR technique

**Fig. 7:**
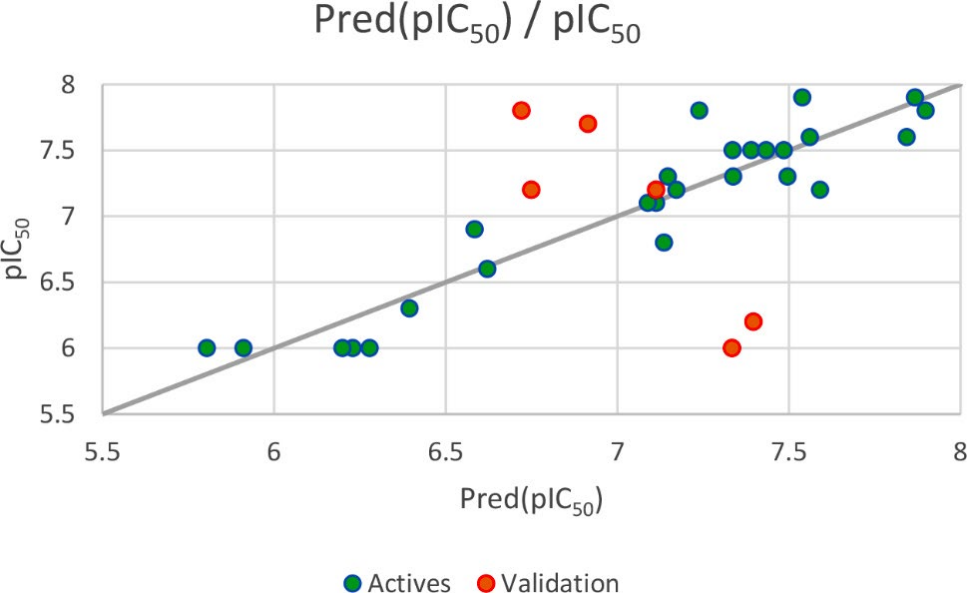
Correlation between the observed and predicted activities using MNLR technique

Based on the set test (*N* = 6), the first regression established by MLR method and illustrated in Fig. 5, results an external validation correlation coefficient (R^2^ext = 0.703) between the observed and predicted activities, with an adjustment coefficient (*R*^*2*^*adjusted* = 0.629) and a minimal root mean square error (*RMSE* = 0.460). The second regression shown in Fig. 6, gives an external validation correlation coefficient (*R*^*2*^*ext* = 0.851) between the observed and predicted activities, with a good adjustment coefficient (*R*^*2*^*adjusted* = 0.814) and a minimal root mean square error (*RMSE* = 0.326). The third regression as presented in Fig. 7, gives an external validation correlation coefficient (*R*^*2*^*ext* = 0.778) between the observed and predicted activities, with a good adjustment coefficient (*R*^*2*^*adjusted* = 0.722) and a minimal root mean square error (*RMSE* = 0.398) and the last regression, results an external validation correlation coefficient of 98.1%. According to ‘Alexander Golbraikh and Alexander Tropsha’ theory, an external validation correlation coefficient greater than 0.6 shows that the established QSAR model is externally validated. Consequently, we can say that the four QSAR models obtained respectively by MLR, PLSR, and MNLR techniques are externally validated because their correlation coefficients are largely higher than 0.6, so the experimental activity can be precisely predicted using one of four established QSAR models including all five of the following descriptors: E lumo, χ, µ, Log P and % [61].

#### Cross-validation

To measure the effectiveness of the QSAR models and examine their reliability, we have applied the cross-validation technique with the leave-one-out procedure, removing each observation at each iteration, so that each observation is tested exactly a once [62, 63]. This technique is applied searching each time a new model of twenty-five compounds (N-1 = 25) and predicting the biological activity of the deleted sample, as noted in Table 6, such that quadratic coefficient q^2^ or (r^2^cv) given by Eq. 6 is greater than 0.5, for that the models will be internally validated [64].

6 $$\:{r}^{2}cv=1-\:\frac{{\sum\:}_{1}^{26}\:\left(Ypred-Yobs\right){\:}^{2}}{{\sum\:}_{1}^{26}\:\left(Yobs-Ymean\right){\:}^{2}}=0.785$$

As:

*Y pred*: the LOO predicted response value, *Y obs*: the observed response value and *Y* mean: is the average of the observed response values.

A high value of *r*^*2*^*cv* = 0.785 (greater than 0.5) indicates that the candidate QSAR model is reliable, robust, and has better internal predictivity. Despite this, ‘Alexander Golbraikh and Alexander Tropsha’s study confirms that the cross-validation technique is essential but not sufficient, because the internal predictive power of the cross-validation procedure tends to be overestimated and the high value of the quadratic coefficient may result from a hazard correlation. For this reason, a Y-randomization test is necessary [61].

**Table 6:** Observed and predicted pIC_50_ values of the training set from the QSAR models

Compounds Number	Observed pIC_50_	Pred pIC_50_ MLR	Pred pIC_50_ PLSR	Pred pIC_50_ MNLR	Pred pIC_50_ CV (LOO)
C1	7.100	7.032	6.652	7.113	6.985
C2	7.100	7.007	6.809	7.089	6.963
C3	7.200	7.229	7.386	7.172	7.246
C5	7.300	6.983	7.166	7.147	6.933
C6	7.600	7.450	7.596	7.561	7.419
C7	7.500	7.303	7.540	7.390	7.240
C8	6.000	5.835	6.267	5.804	5.654
C9	6.000	6.176	6.491	6.229	6.262
C10	7.500	7.569	7.970	7.434	7.623
C11	6.300	6.495	6.645	6.395	6.534
C12	7.800	7.253	7.125	7.239	7.209
C13	6.000	6.079	5.910	5.911	6.108
C14	6.000	6.174	6.259	6.200	6.333
C15	6.000	6.409	6.443	6.279	6.504
C16	6.900	6.715	6.620	6.584	6.690
C17	7.600	7.810	7.631	7.843	7.844
C18	6.600	6.327	6.339	6.621	6.129
C20	6.800	7.129	6.994	7.136	7.190
C21	7.500	7.468	7.363	7.485	7.466
C22	7.900	7.508	7.373	7.539	7.469
C23	7.800	7.874	7.831	7.898	7.888
C25	7.500	7.390	7.255	7.336	7.347
C27	7.200	7.641	7.490	7.591	7.687
C29	7.300	7.364	7.170	7.338	7.370
C31	7.900	7.924	7.684	7.867	7.929
C32	7.300	7.555	7.691	7.496	7.585

#### Validation using the Y-randomization test

To control the robustness of the QSAR model, we have used the Y-randomization test (or permutation test), based on repetitive randomizations of the answer data (Y). Thus, a new model is derived on a new training test of 26 compounds. But to improve the precision of the probability level, a few hundred runs of rerandomized data are usually necessary [65, 66]. The results of the validation using Y-randomization test displayed in Table S3, give the following statistical regression data of random model’s parameters:

$$\begin{aligned}&\text{Average\;r}: 0.421739, \text{Average\;r}^{2}=0.192447, \\&\text{Average\;Q}^{2}_{\text{cv}}=-0.42364,\; \text{and}\;\mathbf{cR}^{2}\mathbf{p}=\textbf{0.766375.}\end{aligned}$$

Although the re-ordered data give much lower *R*^*2*^ than the original data, and randomization constant (**cR**^**2**^**p)** is superior than 0.5, so we can be sure that the previous MLR QSAR model is correct, robust, and is not due to random chance.

#### Applicability domain

**Fig. 8:**
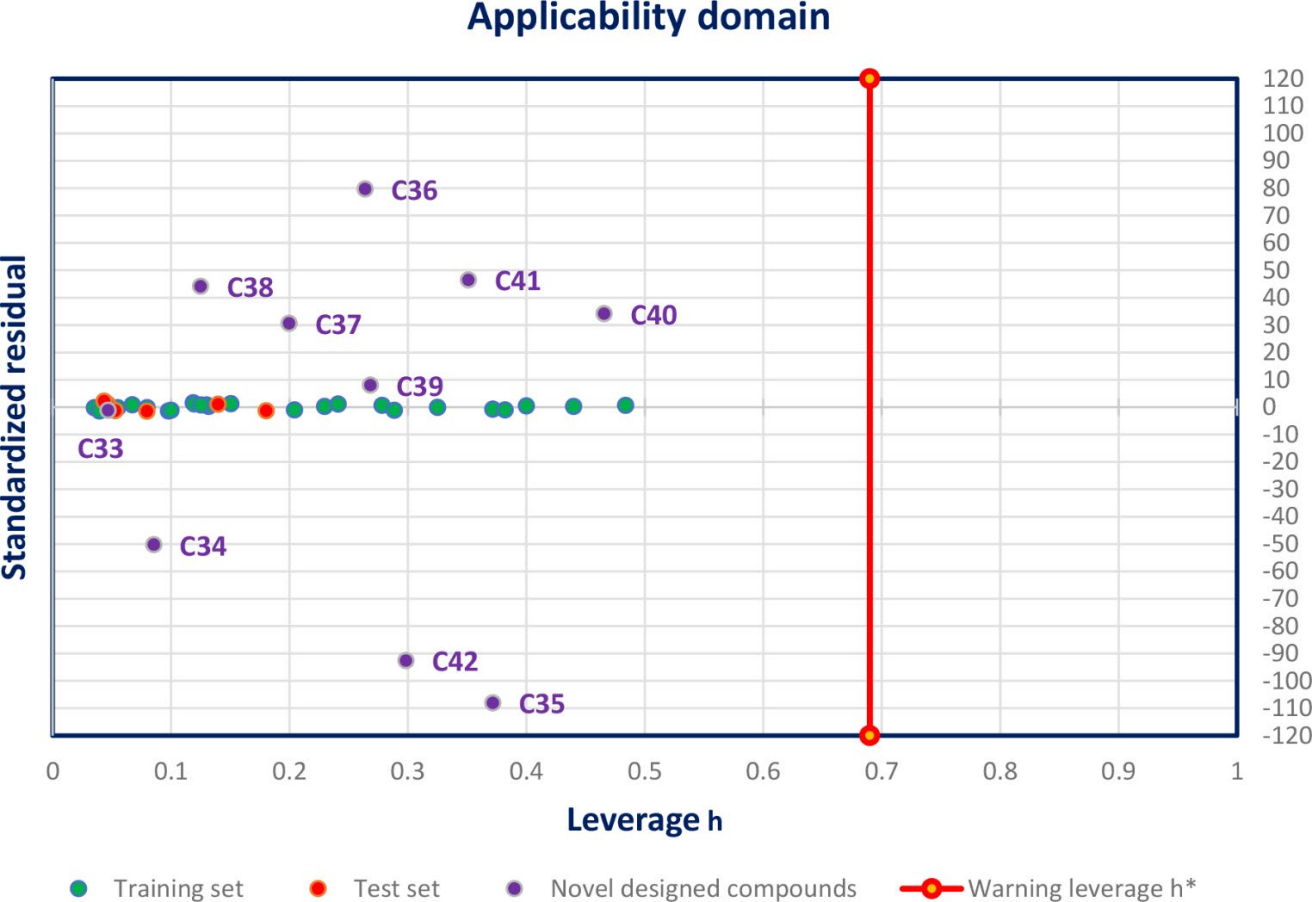
William’s diagram of MLR model established by Eq: (1)

The main objective of the applicability domain (AD) for a predictive classification QSAR model is to define the area of chemical space where the model makes predictions with a given reliability [67]. For this goal, AD was carried out for the best QSAR model established by the MLR technique as given in Eq. 1, based on the leverage analysis illustrated by William diagram (Fig. 8) [68], which is performed with the assistance of SPSS software. An observation with hi > h* significantly affects the performance of the regression and can be classified as an outlier not associated to a reliable prediction. To know, the warning leverage (h*) is defined as h*=3*K/n, and K = *p* + 1, Where n: is the number of the training set, and p: is the number of predictor descriptors [69].

The results show that the leverage values of all chemical compounds in the training and test sets, more than ten newly designed compounds were inferior to the warning leverage h* = 0.69 (*p* = 5, K = 6, *n* = 26) without exception. So, the QSAR MLR model was predicted correctly due to the absence of outliers. Therefore, all compounds are tested in the AD, which mentions that their predicted activities are reliable [70].

### Drug similarity prediction of C22 compound, and novel molecules based on Lipinski, Ghose, Muegge, Veber and Egan rules

To arrive at a drug-like molecule, at least two of the following Lipinski’s rules must be verified: Molecular weight (≤ 500 g/mol) - Log P (High lipophilicity < 5) - Hydrogen bond donors (< 5) - Hydrogen bond acceptors (≤ 10) − 40 ≤ Molar refractive index ≤ 130. The number of rotatable bonds must be less than 10 [71, 72]. In addition, Lipinski’s violations must not exceed 1, otherwise the molecule may have bioavailability problems and a high probability of not being a drug [73]. The molecular descriptors of new designed molecules (C33 to C42) as presented in the Table 7, were calculated using Density Functional Theory (DFT) technique, and then tested with their synthetic accessibility and their drug similarity rules, specifically Lipinski, Ghose, Egan, Veber and Muegge, based on the most active compound, scored C22.

**Table 7:**
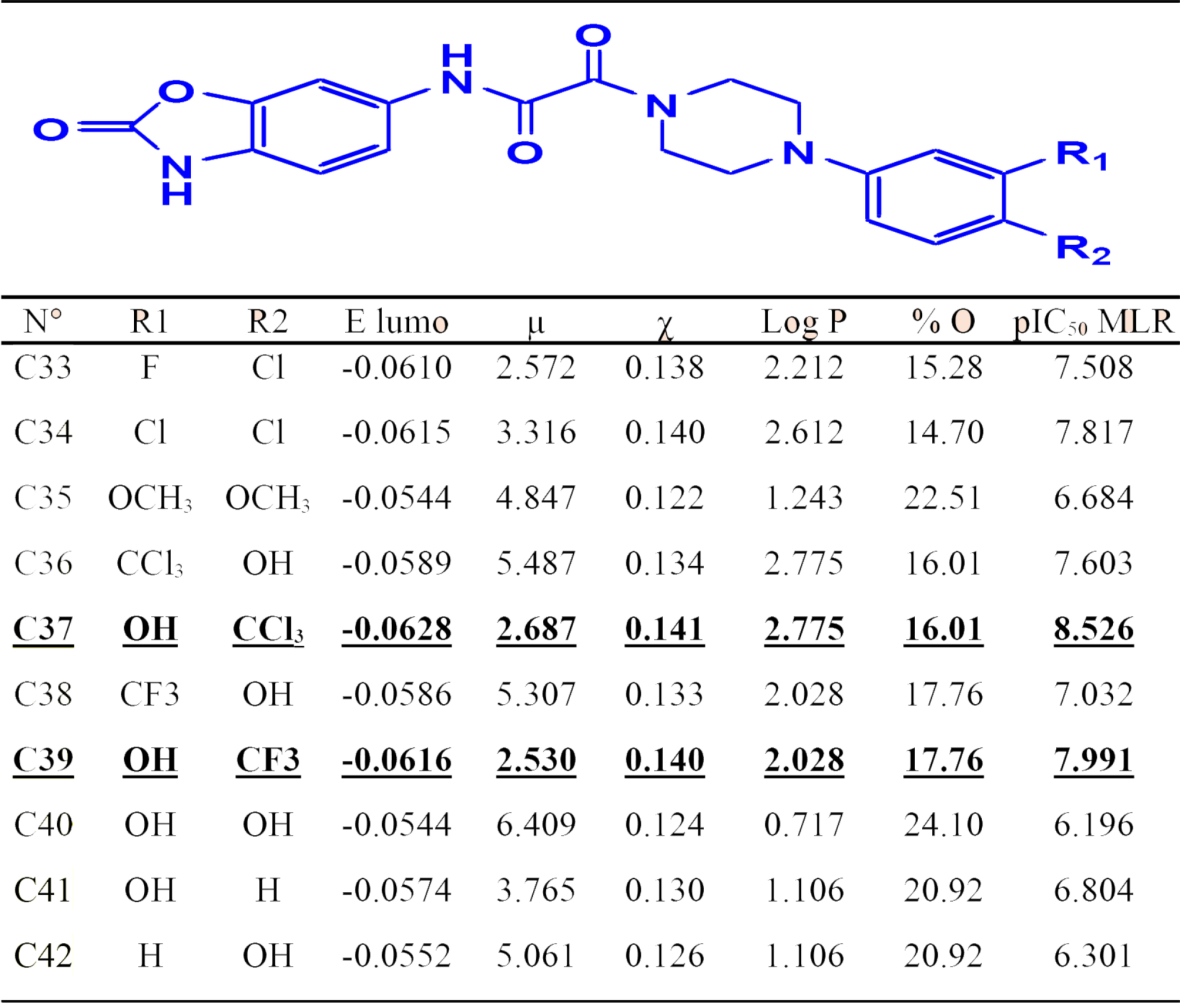
Structural formula of the new studied compounds

The results obtained in Table 8, demonstrate that all ten compounds respect all Lipinski rules, which indicates the absence of oral bioavailability problems. All compounds are easy to synthesize, as their synthetic accessibility values are all about 3. In addition, all designed compounds meet the drug similarity rules except C36, C37 and C40 that do not verify Ghose and Egan drug similarity rules, as presented in Table 9 [74].

**Table 8:** Lipinski rules of the newly designed compounds

Compounds	Physicochemical properties						Lipinski violations
	Molecular weight (g/mol)	Molar refractive index		Log P	H-BA	H-BD	
Rule	≤ 500	40 ≤ MR ≤ 130		< 5	≤ 10	< 5	≤ 1
C33	418.81	112.10		2.28	5	2	Yes
C34	435.26	117.15		1.88	4	2	Yes
C35	426.42	120.11		2.61	6	2	Yes
C36	499.73	128.39		2.51	5	3	Yes
**C37**	**499.73**	**128.39**		**2.43**	**5**	**3**	**Yes**
C38	450.37	114.15		2.13	8	3	Yes
**C39**	**450.37**	**114.15**		**2.02**	**8**	**3**	**Yes**
C40	398.37	111.17		1.71	6	4	Yes
C41	382.37	109.15		1.96	5	3	Yes
C42	382.37	109.15		1.74	5	3	Yes

**Table 9:** Drug likeness prediction of the selected compounds and C22 compound based on Ghose, Muegge, Veber, and Egan rules, and their synthetic accessibility

Compounds	Muegge	Veber	Egan	Ghose	Synthetic accessibility	
C33	Yes	Yes	Yes	Yes	3.23	
C34	Yes	Yes	Yes	Yes	3.26	
C35	Yes	Yes	Yes	Yes	3.50	
C36	Yes	Yes	Yes	NO	3.33	
**C37**	**Yes**	**Yes**	**Yes**	**NO**	**3.33**	
C38	Yes	Yes	Yes	Yes	3.26	
**C39**	**Yes**	**Yes**	**Yes**	**Yes**	**3.28**	
C40	Yes	Yes	NO	Yes	3.30	
C41	Yes	Yes	Yes	Yes	3.25	
C42	Yes	Yes	Yes	Yes	3.21	

### ADMET in silico pharmacokinetics

To identify the new candidate drug with high success level and reduced experimental study duration, new compounds were designed based on the most potent inhibitor scored (C22), as presented in Table 7. This in-silico study predicts the pharmacokinetic features of Adsorption, Distribution, Metabolism, Excretion and Toxicity (ADMET) [75] for ten compounds satisfying all conditions mentioned in Lipinski’s rule, as illustrated in Table 8. According to the results presented in Table 10, all the proposed compounds have a good absorption in the human intestine (IAH more than 70%) [72]. Except for C36, C37, C38 and C39, all the new compounds have a good distribution, as their human distribution volumes are estimated to be higher than − 0.44 Log L/kg, their BBB permeability superior than − 1 Log BB, and their CNS permeability included between − 2 and − 3 Log PS, thus do not penetrate the central nervous system [76]. C36 and C37 compounds, are potential inhibitors of cytochrome CYP450 (2C19), C33 to C39 molecules are possible inhibitors of cytochrome CYP450 (3A4), and C33-34 and C36-39 molecules could be inhibitors of cytochrome CYP450 (2C9). These last inhibitors are the only ones that have a low total clearance value, which means a great success of drug elimination by the organism. Any of these molecules having the AMES toxicity, this last property of ADMET has a major role in drug discovery [73]. Finally, we have succeeded in finding two new molecules, with highest analgesic activities (more than most active compound C22); verifying Lipinski, Muegge, Veber and Egan rules, and all in silico ADMET properties without exception, the first one noted C37 and the second one noted C39, they are predicted as powerful inhibitors of both cytochromes CYP450 (2C9) and CYP450 (3A4), with analgesic activities of pIC_50_ = 8.526 and pIC_50_ = 7.991, respectively.

**Table 10:** ADMET properties prediction of newly engineered compounds

Compounds	Absorption	Distribution			Metabolism							Excretion	Toxicity
	Intestinal absorption (human)	VDss (human)	BBB permeability	CNS permeability	Substrate		Inhibitor					Total Clearance	AMES toxicity
					CYP								
					2D6	3A4	1A2	2C19	2C9	2D6	3A4		
	Numeric (% Absorbed)	Numeric (Log L/kg)	Numeric (Log BB)	Numeric (Log PS)	Categorical (Yes/No)							Numeric (Log ml/min/kg)	Categorical (Yes/No)
C33	84.965	-0.376	-0.91	-2.442	No	Yes	No	No	Yes	No	Yes	-0.041	Not toxic
C34	85.861	-0.244	-0.872	-2.286	No	Yes	No	No	Yes	No	Yes	0.045	Not toxic
C35	78.69	-0.196	-0.917	-2.882	No	Yes	No	No	No	No	Yes	0.667	Not toxic
C36	79.355	-0.455	-1.123	-2.342	No	Yes	No	Yes	Yes	No	Yes	-0.184	Not toxic
**C37**	**79.418**	**-0.447**	**-1.123**	**-2.344**	**No**	**Yes**	**No**	**Yes**	**Yes**	**No**	**Yes**	**-0.125**	**Not toxic**
C38	79.526	-0.535	-1.006	-2.544	No	Yes	No	No	Yes	No	Yes	0.389	Not toxic
**C39**	**79.588**	**-0.529**	**-1.006**	**-2.547**	**No**	**Yes**	**No**	**No**	**Yes**	**No**	**Yes**	**0.414**	**Not toxic**
C40	71.147	-0.025	-0.904	-2.896	No	No	No	No	No	No	No	0.553	Not toxic
C41	77.503	-0.051	-0.709	-2.692	No	No	No	No	No	No	No	0.765	Not toxic
C42	77.44	-0.056	-0.709	-2.694	No	No	No	No	No	No	No	0.575	Not toxic

**Fig. 9:**
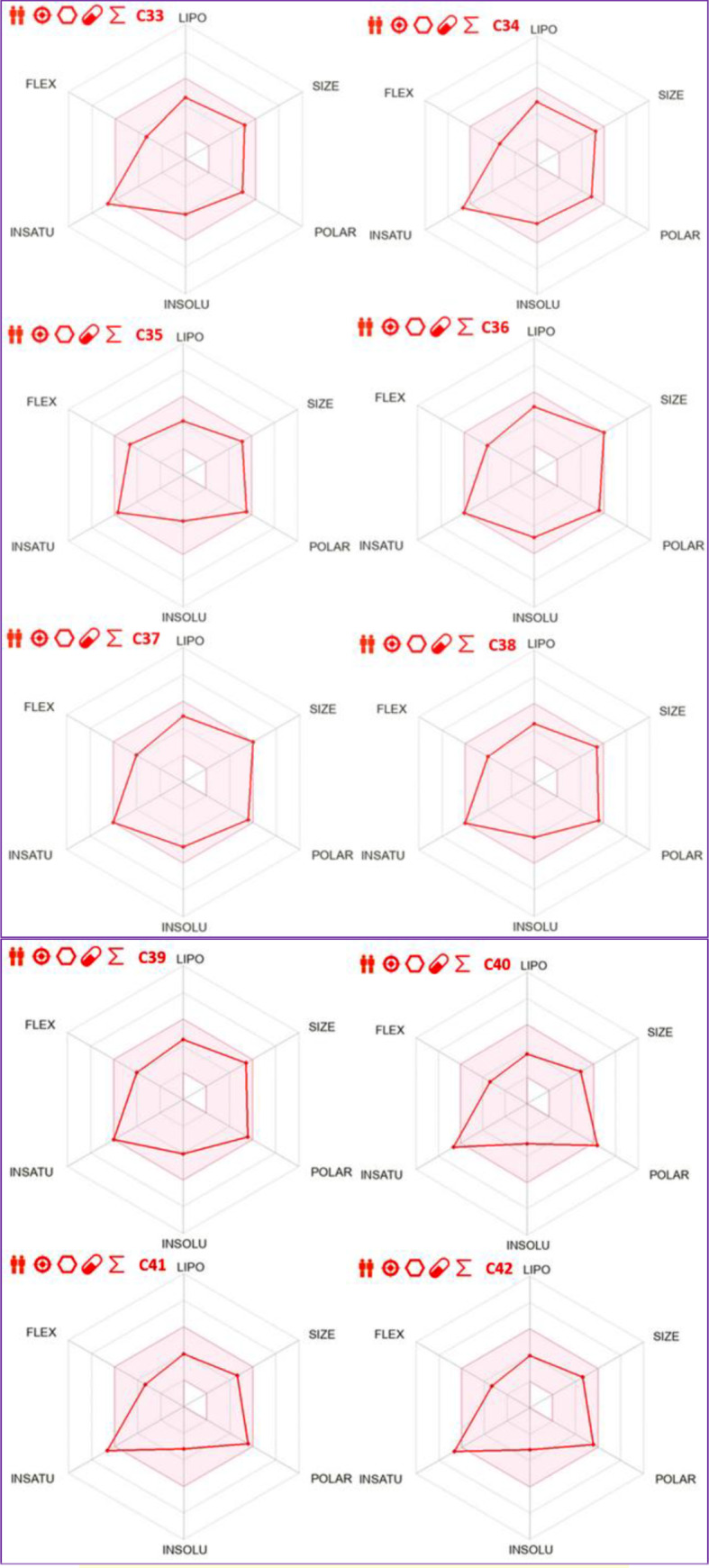
Bioavailability radars for ten designed molecules (C33 to C42)

Additionally, the bioavailability test confirm that all these novel compounds were predicted with an excellent oral bioavailability by the human body, because all tested molecules are part of the pink area of bioavailability radars presented in Fig. 9, as a desired part selected on the basis of the physicochemical parameters of flexibility, saturation, solubility, lipophilicity, polarity and size.

### Molecular docking

**Fig. 10:**
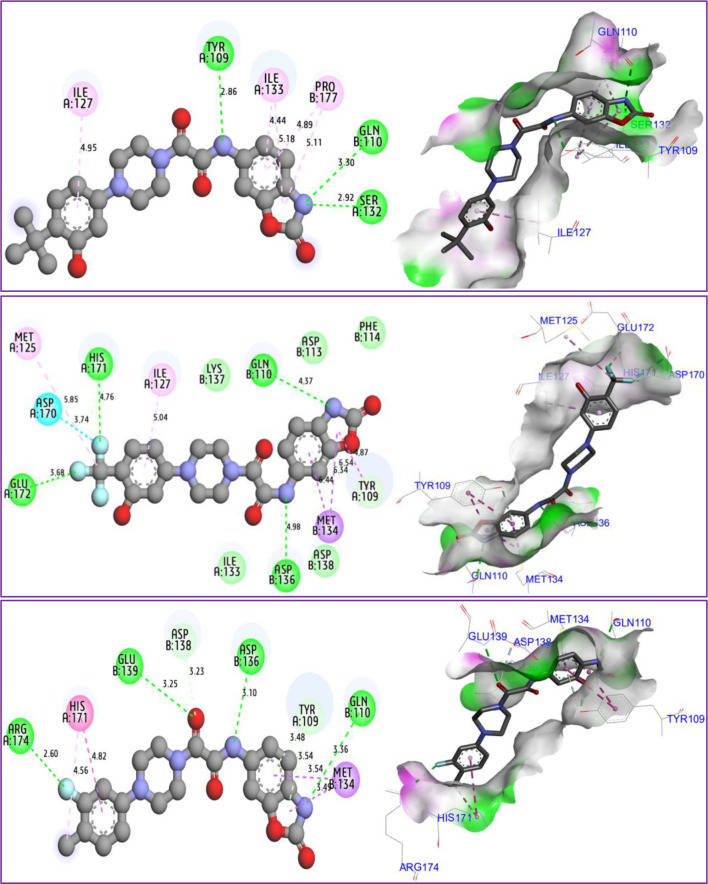
2D and 3D docking positions illustrating the resulted interactions for C37, C39, and C22 phenylpiperazine derivatives, in complex with NMDA receptor coded as 5EWJ.pdb, with binding energies of -8.22, -8.14, and − 7.84 kcal/mol, respectively

**Fig. 11:**
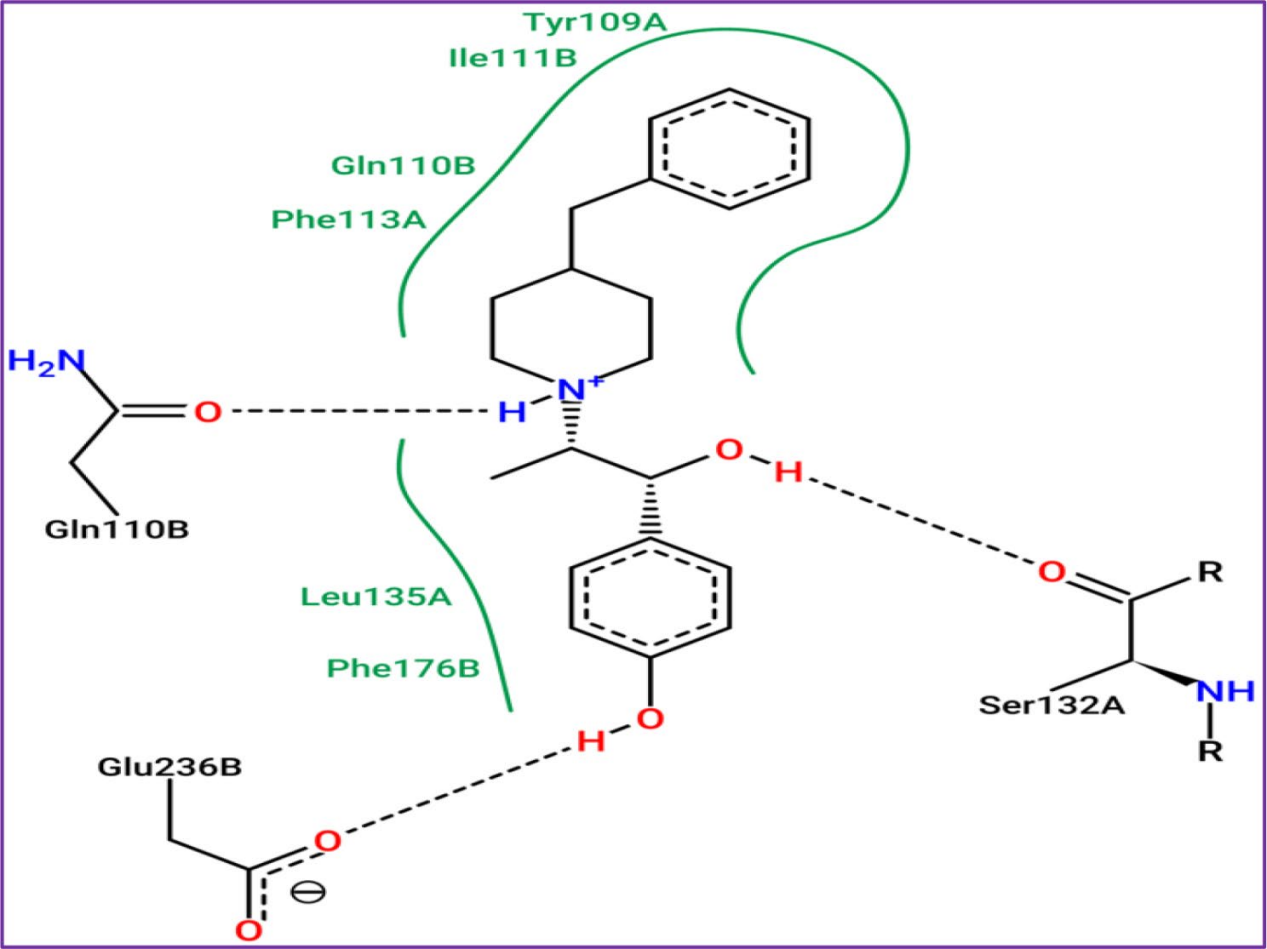
Active sites in A and B chains of NMDA receptor coded by 5EWJ.pdb code

The results of molecular docking simulations shown in Fig. 10, confirm that both novel designed molecules (C37 and C39) were efffectively docked to the active sites of NMDA receptor with lowest possible binding energies in kcal/mol (-8.22 and − 8.14, respectively.), producing common intermolecular interactions, like those detected towards Gln110, Tyr109 amino acids residues (AARs), which were the same chemical bounds detected with the most active compound labeled C22, which was equally docked to the same targeted receptor with a binding energy closer than the previous ones (-7.84 kcal/mol). All other types of intermolecular interactions resulted in each (ligand-5EWJ.pdb protein) complex are presented in Table 11, in which we have equally noticed that both designed molecules (C37 and C39) in addition to the most active ligand (C22) were docked no further than protein target active sites, including Ser132 AAR in A chain and Gln110 AAR in B chain (Fig. 11), which affirm again that the studied ligands were actually docked to the active sites of the protein target, so the processes of molecular docking are successfully validated.

**Table 11:**
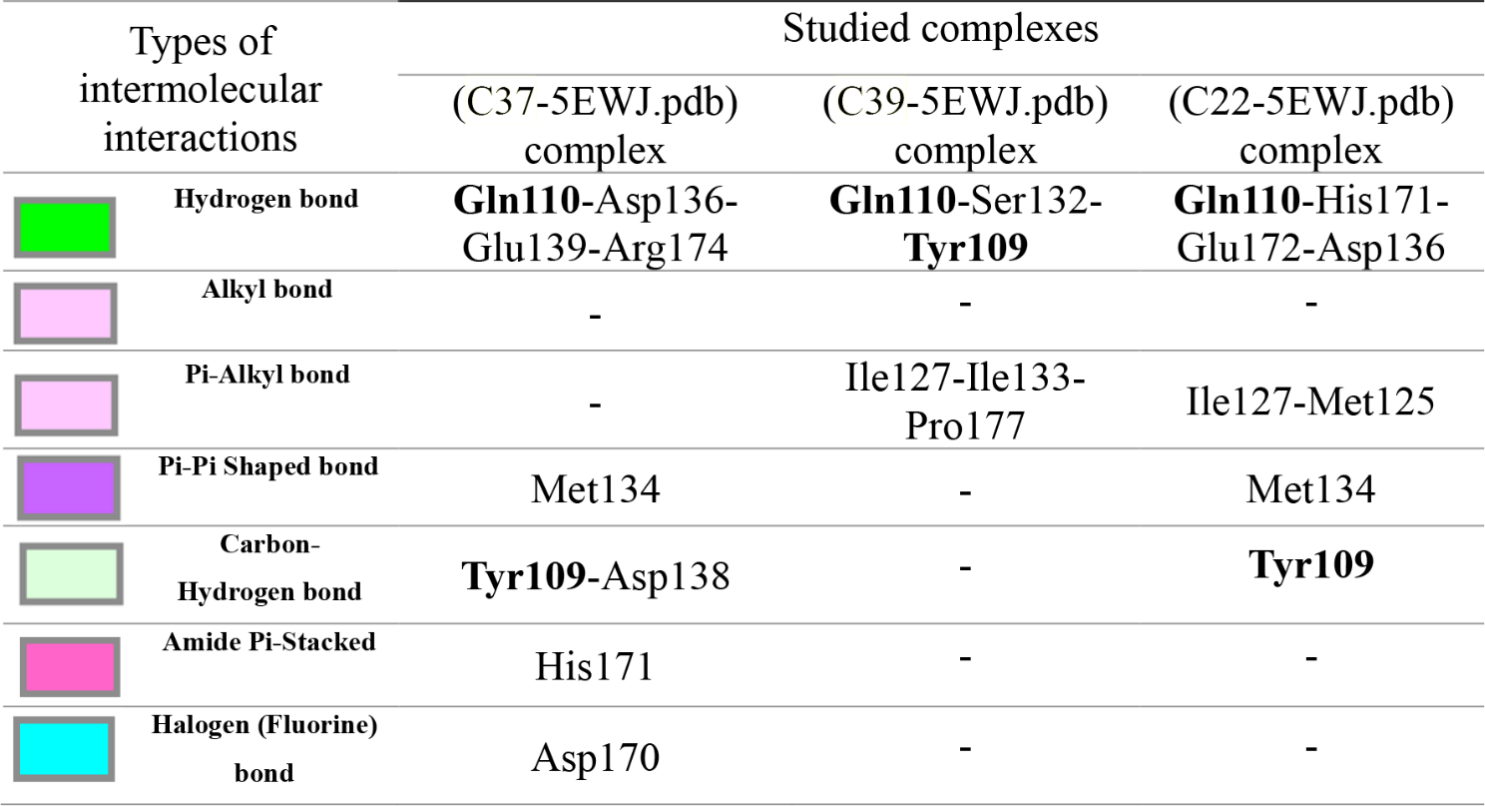
Types of intermolecular interactions produced in three studied complexes

### MD simulation

Drug-receptor complex has to be sufficiently stable over a nano-scaler time range to execute the therapeutic response. As a result, the macromolecular complex underwent a 100 ns MD simulation by using Schrodinger’s Desmond software version 2022.4. The NMDA receptor’s dimeric structure is having of 710 amino acids consisting of 5502 heavy atoms out of overall 10,994 atoms. Structural alterations and RMSD analysis of the macromolecular backbone was executed during the 100 ns simulation to evaluate their thermodynamic stability. The complexed ligand C37 comprising of thirty-two heavy atoms of fifty-eight atoms in total with the presence of seven rotatable bonds. The RMSD value of the NMDA receptor’s backbone was found to fluctuate between 1.5 and 4.0 Å, whereas the bound ligand C37 exhibited some fluctuations within the macromolecular cavity with RMSD value in range of 3.0-5.5 Å followed by a sharp conformational change within the target cavity.

The atoms in a protein or ligand structure might deviate from their initial location, and this can be measured by using their RMSF value. It is an important parameter for determining the flexibility and dynamic behavior of the macromolecular complex. Protein RMSF is important because it may be used to predict protein dynamics and evaluate stability by providing information about the relative flexibility of various regions. MD based evaluation of human NMDA receptor complexed with C37 has concluded that the RMSF for Cα backbone was found to be within 0.6–3.2 Å with couple of exceptions, while for ligand C37 higher RMSF value lies withing the range of 2–4 Å within the target cavity.

The development of hydrophobic contacts, ionic interactions, and hydrogen bonds during an MD simulation are responsible for the thermodynamic permanence of a receptor-ligand complex and it is evaluated by the continuous monitoring of their strengths throughout the simulation for all the three macromolecular complexes. Throughout the simulation ligand C37 was found to be interacting with the NMDA receptor via formation of hydrophobic bonds with the amino acids Tyr109_A, Ile127_A, Tyr128_A, Ile133_A, His134_A, Met134_B, and Pro177_B, whereas amino acid Tyr109_A, Ser132_A, Leu135_A, Lys137_B, and Asp138_B via hydrogen bonding, while residues Ser108_A, Tyr109_A, Ser132_A, Ser136_A, Gln110_B, Asp113_B, Ala135_B, Asp136_B and Asp138_B are found to be interacting via water bridges.

The complexed ligand M7 comprising of thirty-two heavy atoms of forty-nine atoms in total with the presence of seven rotatable bonds. The RMSD value of the receptor’s backbone was found to fluctuate between 1.5 and 3.5 Å, whereas the bound ligand C39 exhibited stable conformation throughout the simulation within the macromolecular cavity with RMSD value in range of 6.0–9.0 Å within the target cavity.

MD based evaluation of NMDA receptor complexed with ligand C39 has concluded that the RMSF for Cα backbone was found to be within 0.8–2.4 Å with couple of exceptions, while for ligand C39 is having RMSF value within the range of 2.0–3.0 Å. indicating its high stability within the target cavity. Throughout the simulation ligand C39 was found to be interacting with NMDA receptor via formation of hydrophobic bonds with the amino acids Tyr109_A, Phe113_A, Ile127_A, Ile133_A, His134_A, and Ala107_B, whereas residues Thr105_A, Gly112_A, Ser129_A, Asp130_A, Gln105_B, Gln110_B, and Asp136_B, Val43, Ala47, Val71, Ile78, Ile90, Val120, and Val167 are found to be interacting via hydrogen bonding, while amino acid Thr105_A, Ser108_A, Arg115_A, Asp130-A, Ser132_A, Ser136_A, Thr76_B, Glu106_B, Asp113_B, Asp136_B and Asp138_BAsn46, Asp49, Glu50, Asp73, Arg76, and Gly77 are found to be interacting via water bridges.

**Fig. 12:**
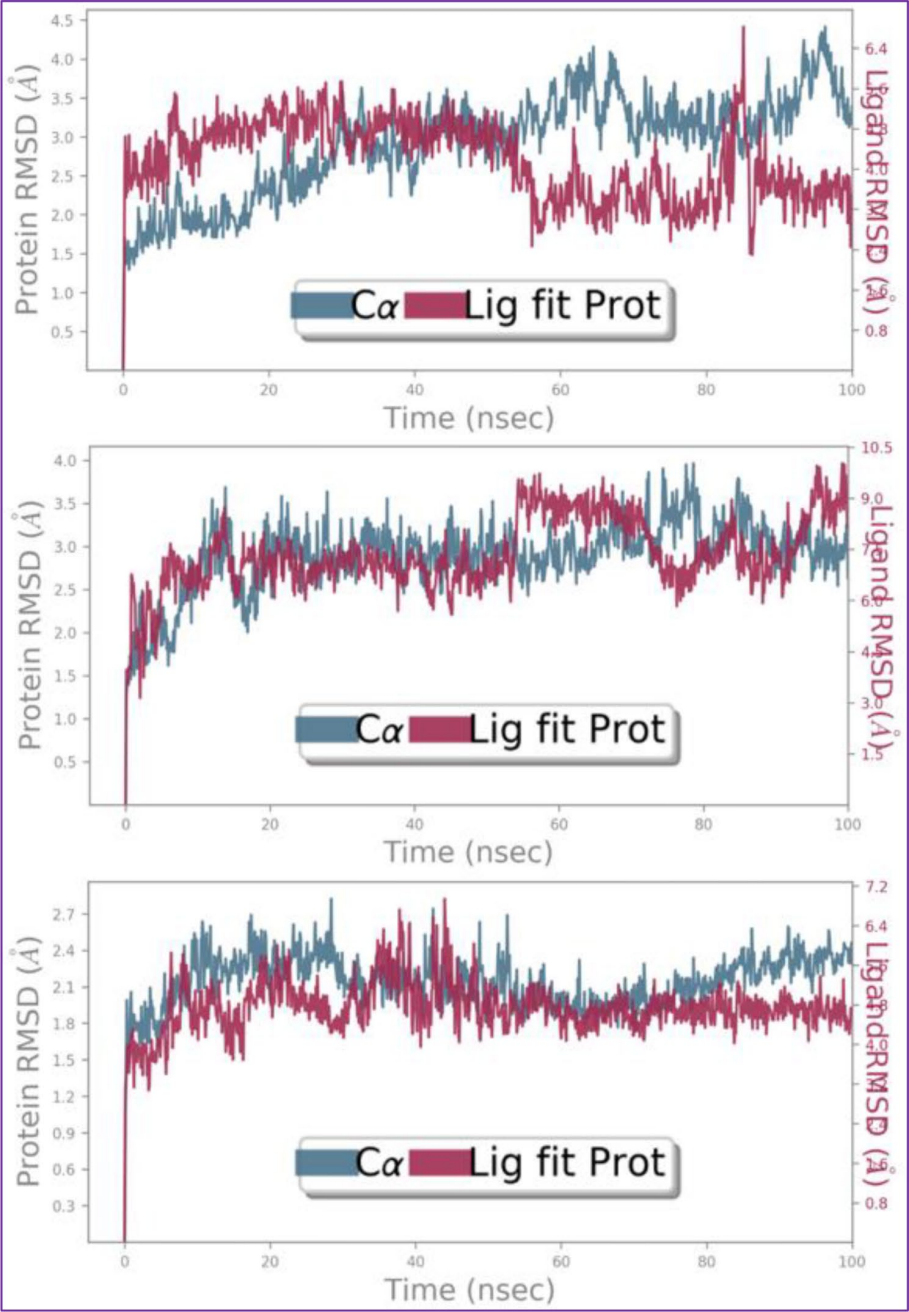
RMSD for Cα chain backbone and complexed ligand for the macromolecular complex of NMDA receptor with ligands C37, C39, and standard drug C22 respectively, detected while executing 100ns MD simulation

The complexed standard drug C22 comprising of twenty-nine heavy atoms of forty-eight atoms in total with the presence of five rotatable bonds. The RMSD value of the NMDA receptor’s backbone was found to fluctuate between 1.5 and 2.7 Å, whereas the bound ligand C22 exhibited stable conformation throughout the simulation within the macromolecular cavity with RMSD value in range of 4.0-6.4 Å within the target cavity. Figure 12 demonstrates the revealed RMSD for macromolecular complexes of NMDA receptor complexed with ligand (a) C37, (b) C39, and standard drug (c) C22.

**Fig. 13:**
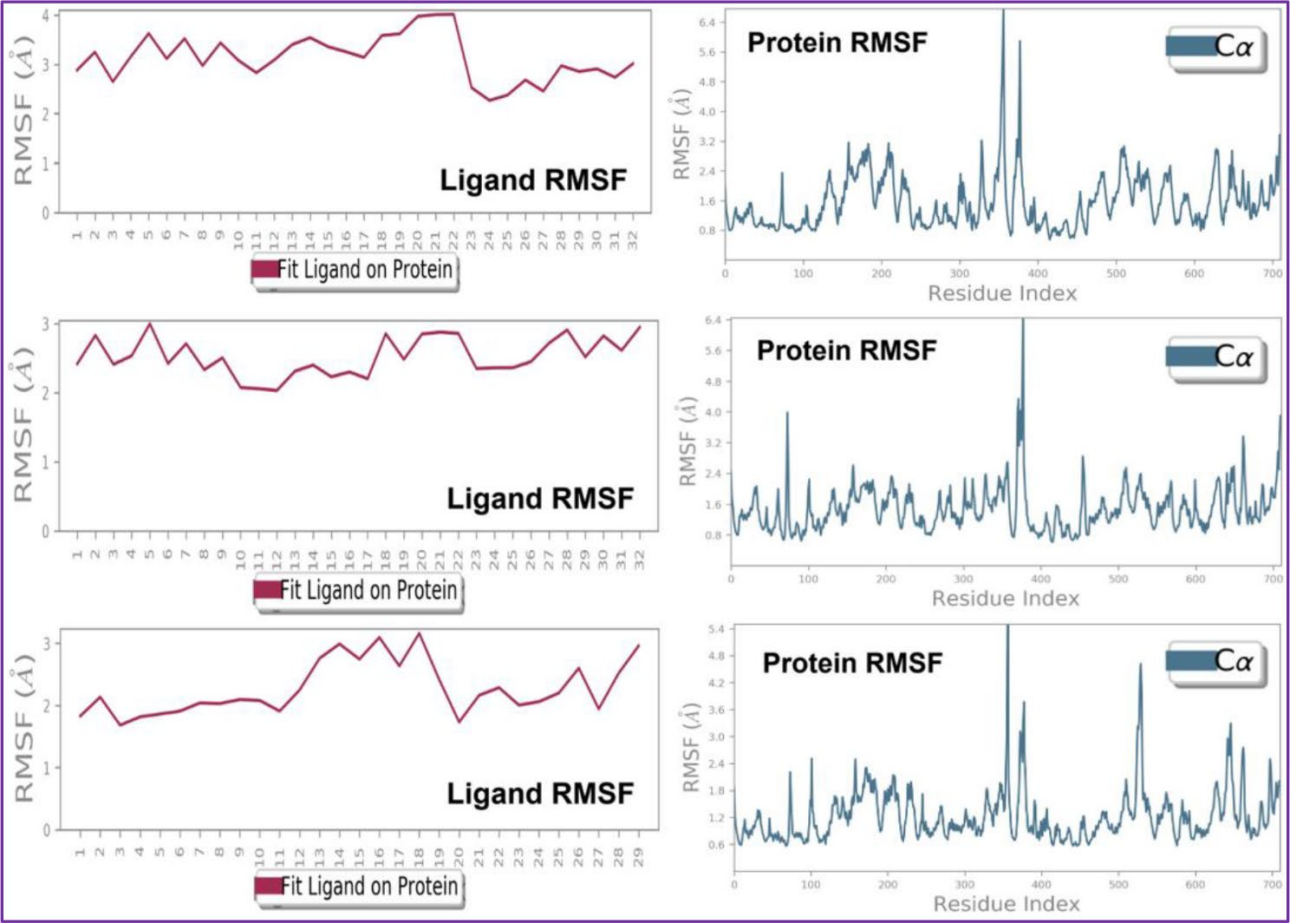
Root mean square fluctuation: Observed RMSF for the macromolecular complex of C37, C39, and standard drug C22 respectively, with human NMDA receptor detected while executing 100ns MD simulation

MD based evaluation of human NMDA receptor complexed with ligand C22 has concluded that the RMSF for Cα backbone was found to be within 0.6–2.4 Å with couple of exceptions, while for ligand C22 is having RMSF value within the range of 2.0–3.0 Å. indicating its high stability within the target cavity. RMSF for macromolecular complexes of human NMDA receptor complexed with ligand (a) C37, (b) C39, and standard drug (c) C22 was depicted in Fig. 13.

**Fig. 14:**
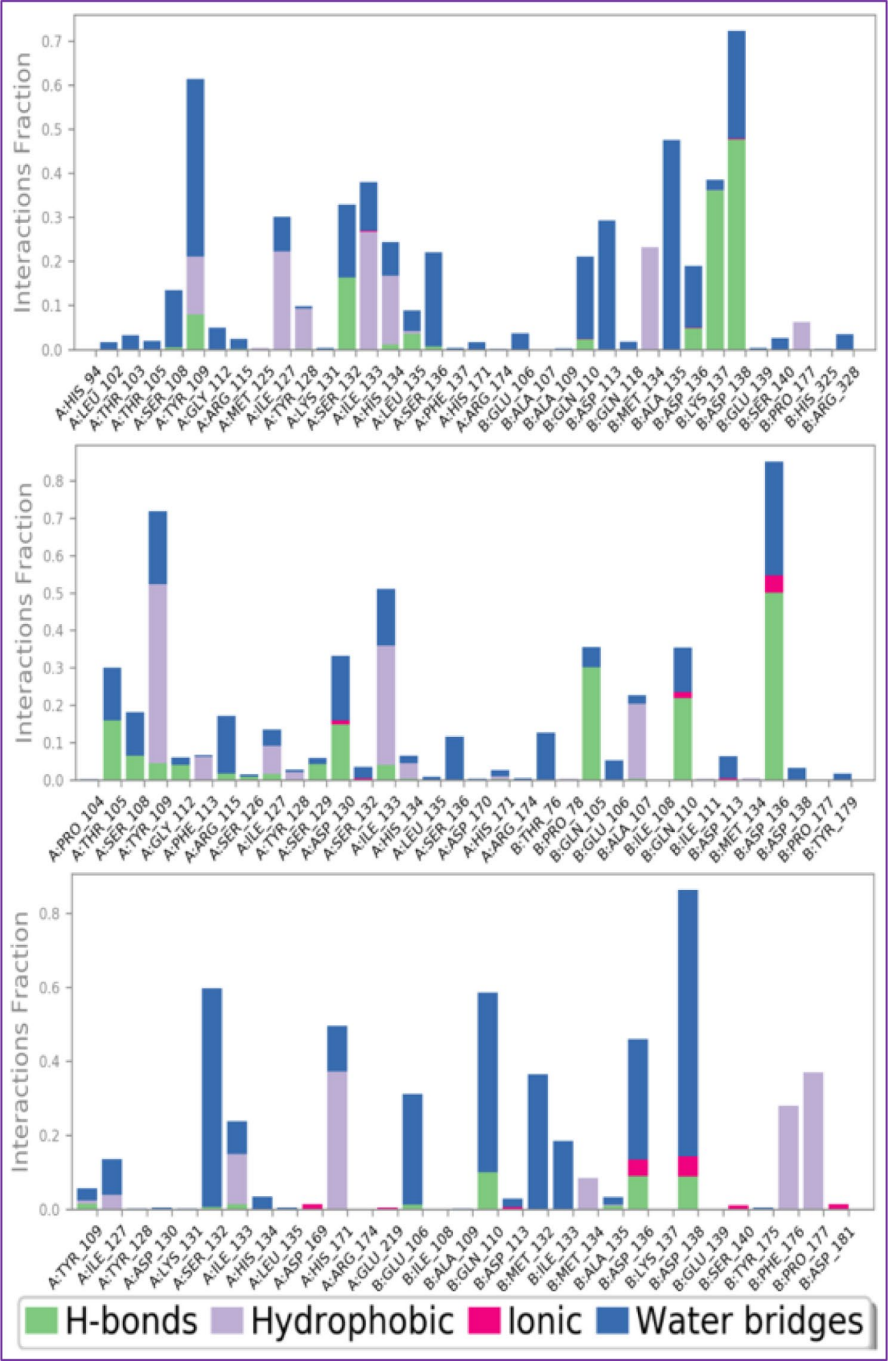
Protein-ligand contacts: Protein-ligand interactions identified between human NMDA receptor complexed with ligands C37, C39, and standard drug C22. The interactions were visualized using different colored bars, with green representing hydrogen bonds, blue representing water bridges, and purple representing hydrophobic interactions

Throughout the simulation ligand C22 was found to be interacting with human NMDA receptor via formation of hydrophobic bonds with the amino acids Ile133_A, His171_A, Met134_B, Phe176_B and Pro177_B, whereas residues Gln110_B, Asp136_B, and Asp138_B are found to be interacting via hydrogen bonding, while amino acid Ile127_A, Ser132_A, His134_A, Glu106_B, Gln110_B, Met132_B, Ile133_B, Asp136_B, and Asp138_B are found to be interacting via water bridges. Figure 14 illustrates the interacting residues of human NMDA receptor with complexed ligand C37, C39, and standard drug C22.

## Conclusions

To concept new analgesic drugs for the treatment of neuropathic pain, four QSAR models were developed and successfully validated using different assessment methods, in which the analgesic activity was significantly affected by the physicochemical, constitutional, and quantum descriptors, in particular: dipole moment, octanol/water partition coefficient, Oxygen mass percentage, electronegativity, and energy of the lowest unoccupied molecular orbital. These results are partially qualified by molecular docking study, as the intermolecular interactions produced by the most active compound (C22) towards the active sites of protein target coded 5EWJ, confirm the production of the chemical bonds with highly electronegativity atoms, such as fluorine and chlorine in meta and para positions, respectively. This is in good agreement with the results developed by the QSAR model, as a noticeable increase in electronegativity, in the presence of an enriched mass percentage of the oxygen element renders the ligand a highly potent analgesic. These results could provide important structural information needed to optimize new good drug candidates for the treatment of neuropathic disease. Among these new drugs, two non-toxic compounds noted C37 and C39, respectively, were predicted to satisfy Lipinski, Muegge, Veber and Egan rules, with excellent ADMET profiles, and very good level of molecular stability towards NMDA receptor. For these reasons, C37 and C39 are strongly recommended to treat neuropathic pain. However, they must be subjected to experimental in vivo and in vitro investigations to examine their safety and efficacy as anti-chronic pain analgesic.

### Electronic supplementary material

Below is the link to the electronic supplementary material.

Supplementary Material 1

## Data Availability

No datasets were generated or analysed during the current study.
