# Supplementary material for: In silico insights into the design of novel NR2B-selective NMDA receptor antagonists: QSAR modeling, ADME-toxicity predictions, molecular docking, and molecular dynamics investigations
Source: BMC Chem. 2024 Jul 31;18(1):142. doi: 10.1186/s13065-024-01248-6 (PMC11293250; doi:10.1186/s13065-024-01248-6)
Supplement: Supplementary file 1 — Supplementary Material 1 [file 13065_2024_1248_MOESM1_ESM.docx]

# In silico insights into the design of novel NR2B-selective NMDA receptor Antagonists: QSAR Modeling, ADME-Toxicity Predictions, Molecular Docking, and Molecular Dynamics Investigations.

Mohamed El fadili^1,*^, Mohammed Er-rajy^1^, Somdutt Mujwar^2^, Ajala Abduljalil^3^, Rachid Bouzammit^4^, Mohammed Kara^5^, Hatem A. Abuelizz^6^, Sara Er-rahmani^7^ and Menana Elhallaoui^1^.

1. LIMAS Laboratory, Faculty of Sciences Dhar El Mahraz, Sidi Mohamed Ben Abdellah University, Fez 30000, Morocco;
2. Chitkara College of Pharmacy, Chitkara University, Rajpura-140401, Punjab, India;
3. Chemistry Department, Ahmadu Bello University, Zaria, Nigeria;
4. Engineering Laboratory of Organometallic, Molecular Materials and Environment (LIMOME), Faculty of Sciences Dhar El Mahraz, Sidi Mohamed Ben Abdellah University, 30000 Fez, Morocco;
5. Laboratory of Biotechnology, Conservation and Valorization of Naturals Resources, Faculty of Sciences Dhar El Mahraz, Sidi Mohamed Ben Abdellah University, Fez 30000, Morocco;
6. Department of pharmaceutical chemistry, college of pharmacy, King Saud University, Riyadh, Saudi Arabia;
7. Dipartimento di Chimica, Università di Torino, 10125 Torino, Italy ;

* Corresponding author: [mohamed.elfadili@usmba.ac.ma](mailto:mohamed.elfadili@usmba.ac.ma)

**Table S1.** The values of selected descriptors for the 32 molecules

| **N°** | **E lumo** | **E homo** | **E gap** | **ƞ** | **X** | **µ** | **d** | **Log P** | **K** | **P** | **T** | **% C** | **% H** | **% O** |
| --- | --- | --- | --- | --- | --- | --- | --- | --- | --- | --- | --- | --- | --- | --- |
| C1 | - 0.0435 | - 0.2305 | 0.187 | 0.093 | 0.137 | 3.1181 | 1.266 | 2.508 | 686 310.535 | -1 371 429.744 | -685 435.020 | 67.84 | 5.99 | 9.51 |
| C2 | - 0.0441 | - 0.2309 | 0.187 | 0.093 | 0.137 | 3.5101 | 1.313 | 2.666 | 747 891.085 | -1 494 345.722 | -747 105.031 | 64.40 | 5.40 | 9.03 |
| C3 | - 0.0456 | - 0.2326 | 0.187 | 0.094 | 0.139 | 4.6986 | 1.335 | 3.066 | 972 466.445 | -1 944 427.097 | -972 007.885 | 61.54 | 5.16 | 8.63 |
| C4 | - 0.0612 | - 0.2212 | 0.160 | 0.080 | 0.141 | 3.8193 | 1.265 | 2.011 | 745 962.867 | -1 491 770.268 | - 745 833.850 | 69.41 | 5.82 | 13.21 |
| C5 | - 0.0620 | - 0.2219 | 0.160 | 0.080 | 0.142 | 2.7047 | 1.308 | 2.169 | 807 535.065 | -1 615 326.161 | -807 556.624 | 66.13 | 5.29 | 12.58 |
| C6 | - 0.0625 | - 0.2225 | 0.160 | 0.080 | 0.142 | 2.3047 | 1.330 | 2.569 | 1 032 494.984 | -2 064 892.607 | -1 032 423.164 | 63.40 | 5.07 | 12.06 |
| C7 | - 0.0524 | - 0.2132 | 0.161 | 0.080 | 0.133 | 6.1292 | 1.349 | 2.491 | 854 023.234 | -1 708 738.772 | -854 285.109 | 66.66 | 5.34 | 16.14 |
| C8 | - 0.0641 | - 0.2236 | 0.159 | 0.080 | 0.144 | 2.8998 | 1.374 | 1.389 | 1 042 452.024 | -2 084 989.325 | -1 042 401.229 | 63.40 | 5.07 | 12.06 |
| C9 | - 0.0663 | - 0.2080 | 0.142 | 0.071 | 0.137 | 1.4317 | 1.393 | 1.732 | 1 018 130.074 | -2 036 063.200 | -1 018 026.869 | 62.58 | 4.73 | 12.51 |
| C10 | - 0.0531 | - 0.2135 | 0.160 | 0.080 | 0.133 | 6.4008 | 1.414 | 2.891 | 1 079 121.581 | -2 157 875.754 | -1 079 203.243 | 60.95 | 4.87 | 15.46 |
| C11 | - 0.0559 | - 0.2156 | 0.160 | 0.080 | 0.136 | 4.6633 | 1.462 | 1.711 | 1 089 342.243 | -2 178 217.214 | -1 089 137.602 | 57.90 | 4.62 | 15.43 |
| C12 | - 0.0593 | - 0.2115 | 0.152 | 0.076 | 0.135 | 2.9720 | 1.486 | 2.054 | 1 064 783.166 | -2 129 510.762 | -1 064 781.952 | 56.93 | 4.28 | 15.97 |
| C13 | - 0.0550 | - 0.2088 | 0.154 | 0.077 | 0.132 | 1.5003 | 1.431 | 1.647 | 1 042 224.308 | -2 084 833.186 | -1 042 432.273 | 60.23 | 4.80 | 12.03 |
| C14 | - 0.0566 | - 0.1993 | 0.143 | 0.071 | 0.128 | 1.5991 | 1.393 | 2.064 | 1 066 429.484 | -2 133 213.118 | -1 066 802.365 | 61.09 | 5.13 | 11.63 |
| C15 | - 0.0562 | - 0.2062 | 0.150 | 0.075 | 0.131 | 4.4962 | 1.421 | 1.496 | 778 197.823 | -1 556 604.692 | -778 171.983 | 62.29 | 4.95 | 17.47 |
| C16 | - 0.0577 | - 0.2083 | 0.151 | 0.075 | 0.133 | 3.4828 | 1.467 | 1.654 | 839 830.122 | -1 680 057.272 | -839 873.754 | 59.37 | 4.46 | 16.65 |
| C17 | - 0.0611 | - 0.2187 | 0.158 | 0.079 | 0.140 | 2.3928 | 1.491 | 2.417 | 987 227.946 | -1 974 930.951 | -987 674.081 | 55.30 | 3.94 | 14.73 |
| C18 | - 0.0545 | - 0.1936 | 0.139 | 0.070 | 0.124 | 5.3264 | 1.407 | 1.370 | 849 216.309 | -1 698 470.903 | -849 222.270 | 60.60 | 5.09 | 20.18 |
| C19 | - 0.0596 | - 0.2131 | 0.154 | 0.077 | 0.136 | 2.9733 | 1.512 | 1.812 | 901 559.019 | -1 802 579.297 | -901 596.040 | 56.72 | 4.01 | 15.91 |
| C20 | - 0.0604 | - 0.2181 | 0.158 | 0.079 | 0.139 | 3.0154 | 1.512 | 1.812 | 901 532.939 | -1 802 776.752 | -901 594.539 | 56.72 | 4.01 | 15.91 |
| C21 | - 0.0601 | - 0.2162 | 0.156 | 0.078 | 0.138 | 2.9123 | 1.529 | 2.212 | 1 125 979.272 | -2 252 652.314 | -1 126 478.301 | 54.49 | 3.85 | 15.28 |
| C22 | - 0.0610 | - 0.2155 | 0.154 | 0.077 | 0.138 | 2.5724 | 1.529 | 2.212 | 1 126 457.913 | -2 253 020.354 | -1 126 506.753 | 54.49 | 3.85 | 15.28 |
| C23 | - 0.0630 | - 0.2199 | 0.157 | 0.078 | 0.141 | 2.3379 | 1.531 | 2.575 | 1 048 783.590 | -2 097 818.268 | -1 049 350.239 | 53.10 | 3.57 | 14.15 |
| C24 | - 0.0562 | - 0.1977 | 0.142 | 0.071 | 0.127 | 4.9691 | 1.449 | 1.528 | 910 728.654 | -1 821 586.345 | -910 944.149 | 57.97 | 4.62 | 19.30 |
| C25 | - 0.0599 | - 0.1997 | 0.140 | 0.070 | 0.130 | 2.0747 | 1.432 | 2.373 | 1 089 518.203 | -2 177 963.910 | -1 089 153.555 | 57.90 | 4.62 | 15.43 |
| C26 | - 0.0601 | - 0.2093 | 0.149 | 0.075 | 0.135 | 3.5384 | 1.432 | 2.373 | 1 089 103.231 | - 2 178 115.902 | -1 089 145.422 | 57.90 | 4.62 | 15.43 |
| C27 | - 0.0569 | - 0.2161 | 0.159 | 0.080 | 0.137 | 3.5396 | 1.432 | 2.373 | 1 089 134.342 | - 2 177 951.634 | -1 089 114.080 | 57.90 | 4.62 | 15.43 |
| C28 | - 0.0594 | - 0.2073 | 0.148 | 0.074 | 0.133 | 2.6762 | 1.432 | 2.373 | 1 089 136.388 | - 2 178 423.348 | -1 089 166.708 | 57.90 | 4.62 | 15.43 |
| C29 | - 0.0590 | - 0.2127 | 0.154 | 0.077 | 0.136 | 2.6287 | 1.455 | 2.131 | 925 593.260 | - 1 851 437.187 | -925 933.697 | 57.69 | 4.36 | 15.37 |
| C30 | - 0.0571 | - 0.2162 | 0.159 | 0.080 | 0.137 | 4.4500 | 1.455 | 2.131 | 925 717.641 | - 1 852 146.069 | -925 889.442 | 57.69 | 4.36 | 15.37 |
| C31 | - 0.0604 | - 0.2151 | 0.155 | 0.077 | 0.138 | 2.1664 | 1.473 | 2.531 | 1 150 486.960 | -2 301 319.226 | -1 150 858.034 | 55.50 | 4.19 | 14.79 |
| C32 | - 0.0585 | - 0.2171 | 0.159 | 0.079 | 0.138 | 4.1307 | 1.473 | 2.531 | 1 150 531.027 | -2 301 482.041 | -1 150 808.603 | 55.50 | 4.19 | 14.79 |

**Table S2.** Correlation matrix: (Pearson r)

| **Variables** | **E lumo** | **E homo** | **E gap** | **ƞ** | **X** | **µ** | **d** | **Log P** | **K** | **P** | **T** | **% C** | **% H** | **% O** |  |
| --- | --- | --- | --- | --- | --- | --- | --- | --- | --- | --- | --- | --- | --- | --- | --- |
| E lumo | 1 |  |  |  |  |  |  |  |  |  |  |  |  |  |  |
| E homo | -0.287 | 1 |  |  |  |  |  |  |  |  |  |  |  |  |  |
| E gap | 0.672 | **-0.902** | 1 |  |  |  |  |  |  |  |  |  |  |  |  |
| ƞ | 0.667 | **-0.904** | **0.998** | 1 |  |  |  |  |  |  |  |  |  |  |  |
| X | -0.292 | -0.831 | 0.510 | 0.515 | 1 |  |  |  |  |  |  |  |  |  |  |
| µ | 0.470 | 0.023 | 0.189 | 0.200 | -0.290 | 1 |  |  |  |  |  |  |  |  |  |
| d | -0.432 | 0.355 | -0.467 | -0.469 | -0.105 | -0.160 | 1 |  |  |  |  |  |  |  |  |
| Log P | 0.337 | -0.525 | 0.556 | 0.547 | 0.327 | 0.071 | -0.180 | 1 |  |  |  |  |  |  |  |
| K | -0.440 | 0.204 | -0.360 | -0.355 | 0.060 | -0.273 | 0.567 | 0.206 | 1 |  |  |  |  |  |  |
| P | 0.440 | -0.205 | 0.361 | 0.356 | -0.060 | 0.273 | -0.568 | -0.205 | **-1** | 1 |  |  |  |  |  |
| T | 0.441 | -0.205 | 0.361 | 0.356 | -0.060 | 0.273 | -0.568 | -0.205 | **-1** | **1** | 1 |  |  |  |  |
| % C | 0.360 | -0.271 | 0.370 | 0.369 | 0.060 | 0.211 | **-0.945** | -0.021 | -0.673 | 0.673 | 0.673 | 1 |  |  |  |
| % H | 0.513 | -0.189 | 0.374 | 0.376 | -0.105 | 0.260 | **-0.961** | 0.029 | -0.587 | 0.588 | 0.588 | **0.947** | 1 |  |  |
| % O | -0.334 | 0.717 | -0.706 | -0.700 | -0.519 | 0.370 | 0.571 | -0.461 | 0.111 | -0.112 | -0.112 | -0.444 | -0.441 | 1 | |

**Table S3.** Calculated results using Y-randomization test.

| **Model** | ***R*** | ***R^2*** | ***Q^2*** | **Model** | ***R*** | ***R^2*** | ***Q^2*** |
| --- | --- | --- | --- | --- | --- | --- | --- |
| **Original** | **0.927604367** | **0.860449862** | **0.785267191** | Random 51 | 0.401505 | 0.161206 | -0.54535 |
| Random 1 | 0.285853902 | 0.081712453 | -0.603329982 | Random 52 | 0.269116 | 0.072423 | -0.62871 |
| Random 2 | 0.381024869 | 0.145179951 | -0.551695902 | Random 53 | 0.286199 | 0.08191 | -0.93703 |
| Random 3 | 0.561758014 | 0.315572066 | -0.150155527 | Random 54 | 0.301718 | 0.091034 | -0.6728 |
| Random 4 | 0.535473046 | 0.286731383 | -0.06268153 | Random 55 | 0.407573 | 0.166116 | -0.54452 |
| Random 5 | 0.520721391 | 0.271150768 | -0.495240817 | Random 56 | 0.404192 | 0.163371 | -0.36533 |
| Random 6 | 0.306998365 | 0.094247996 | -0.675178071 | Random 57 | 0.309689 | 0.095907 | -0.68111 |
| Random 7 | 0.643279824 | 0.413808932 | -0.204390458 | Random 58 | 0.320035 | 0.102422 | -0.48056 |
| Random 8 | 0.747535218 | 0.558808903 | 0.298129824 | Random 59 | 0.507922 | 0.257985 | -0.21931 |
| Random 9 | 0.601588935 | 0.361909246 | -0.13894899 | Random 60 | 0.379511 | 0.144028 | -0.45719 |
| Random 10 | 0.223851889 | 0.050109668 | -0.517476825 | Random 61 | 0.142184 | 0.020216 | -0.57003 |
| Random 11 | 0.637965355 | 0.406999794 | -0.028013454 | Random 62 | 0.48203 | 0.232353 | -0.29611 |
| Random 12 | 0.409463984 | 0.167660754 | -0.465492293 | Random 63 | 0.47588 | 0.226462 | -0.54172 |
| Random 13 | 0.294139258 | 0.086517903 | -0.496936306 | Random 64 | 0.337277 | 0.113756 | -0.42381 |
| Random 14 | 0.379083984 | 0.143704667 | -0.644861478 | Random 65 | 0.624736 | 0.390296 | -0.04424 |
| Random 15 | 0.34902507 | 0.121818499 | -0.546445978 | Random 66 | 0.569771 | 0.324638 | -0.28118 |
| Random 16 | 0.343348085 | 0.117887908 | -0.444813169 | Random 67 | 0.446786 | 0.199618 | -0.50928 |
| Random 17 | 0.274378593 | 0.075283612 | -0.706438753 | Random 68 | 0.322718 | 0.104147 | -0.61069 |
| Random 18 | 0.403006994 | 0.162414637 | -0.473288214 | Random 69 | 0.507872 | 0.257934 | -0.27347 |
| Random 19 | 0.428864576 | 0.183924825 | -0.352537561 | Random 70 | 0.367872 | 0.13533 | -0.42395 |
| Random 20 | 0.495354652 | 0.245376231 | -0.237876032 | Random 71 | 0.492969 | 0.243019 | -0.50153 |
| Random 21 | 0.228110074 | 0.052034206 | -0.565906349 | Random 72 | 0.474499 | 0.22515 | -0.07873 |
| Random 22 | 0.436142527 | 0.190220304 | -0.416580138 | Random 73 | 0.365948 | 0.133918 | -0.34973 |
| Random 23 | 0.268015305 | 0.071832204 | -0.52087345 | Random 74 | 0.539025 | 0.290548 | -0.05006 |
| Random 24 | 0.550327553 | 0.302860416 | -0.013216948 | Random 75 | 0.39259 | 0.154127 | -0.52264 |
| Random 25 | 0.604399148 | 0.36529833 | -0.142845493 | Random 76 | 0.544386 | 0.296356 | -0.24762 |
| Random 26 | 0.393015566 | 0.154461235 | -0.451830971 | Random 77 | 0.258235 | 0.066685 | -0.94053 |
| Random 27 | 0.524585747 | 0.275190206 | -0.173367777 | Random 78 | 0.441677 | 0.195079 | -0.33608 |
| Random 28 | 0.384464537 | 0.14781298 | -0.502113811 | Random 79 | 0.546911 | 0.299112 | -0.23528 |
| Random 29 | 0.197485345 | 0.039000462 | -0.818592789 | Random 80 | 0.495295 | 0.245317 | -0.30015 |
| Random 30 | 0.273170312 | 0.074622019 | -0.719090724 | Random 81 | 0.294121 | 0.086507 | -0.54059 |
| Random 31 | 0.327813032 | 0.107461384 | -0.676068631 | Random 82 | 0.191609 | 0.036714 | -0.64752 |
| Random 32 | 0.497350387 | 0.247357407 | -0.255076933 | Random 83 | 0.344031 | 0.118357 | -0.71433 |
| Random 33 | 0.596651623 | 0.35599316 | -0.00967665 | Random 84 | 0.402319 | 0.16186 | -0.48952 |
| Random 34 | 0.598245505 | 0.357897684 | -0.112614793 | Random 85 | 0.498739 | 0.248741 | -0.4401 |
| Random 35 | 0.406471057 | 0.16521872 | -0.308234724 | Random 86 | 0.237633 | 0.056469 | -0.45285 |
| Random 36 | 0.35202708 | 0.123923065 | -0.698337868 | Random 87 | 0.563079 | 0.317058 | -0.19573 |
| Random 37 | 0.556132043 | 0.309282849 | -0.103182004 | Random 88 | 0.309376 | 0.095713 | -0.58632 |
| Random 38 | 0.26680591 | 0.071185394 | -0.742814365 | Random 89 | 0.532517 | 0.283575 | -0.41257 |
| Random 39 | 0.504847581 | 0.254871081 | -0.462029547 | Random 90 | 0.456336 | 0.208242 | -0.35008 |
| Random 40 | 0.519499461 | 0.26987969 | -0.36109994 | Random 91 | 0.402274 | 0.161824 | -0.30292 |
| Random 41 | 0.606156416 | 0.367425601 | -0.38072965 | Random 92 | 0.443241 | 0.196463 | -0.307 |
| Random 42 | 0.424971279 | 0.180600588 | -0.854401069 | Random 93 | 0.422168 | 0.178226 | -0.49953 |
| Random 43 | 0.316905414 | 0.100429042 | -0.715387196 | Random 94 | 0.544568 | 0.296554 | -0.4411 |
| Random 44 | 0.429701583 | 0.184643451 | -0.408123956 | Random 95 | 0.469741 | 0.220657 | -0.64188 |
| Random 45 | 0.583061004 | 0.339960135 | -0.019429293 | Random 96 | 0.318531 | 0.101462 | -0.51119 |
| Random 46 | 0.422703061 | 0.178677878 | -0.290391372 | Random 97 | 0.405593 | 0.164505 | -0.47301 |
| Random 47 | 0.235339461 | 0.055384662 | -0.939495485 | Random 98 | 0.462881 | 0.214259 | -0.36372 |
| Random 48 | 0.448053847 | 0.20075225 | -0.334872445 | Random 99 | 0.41472 | 0.171993 | -0.33639 |
| Random 49 | 0.611337809 | 0.373733916 | 0.012016096 | Random 100 | 0.399141 | 0.159313 | -0.34738 |
| Random 50 | 0.258675994 | 0.06691327 | -0.75991627 |  |  |  |  |
